# Assessment of Worldwide Acute Kidney Injury, Renal Angina and Epidemiology in Critically Ill Children (AWARE): study protocol for a prospective observational study

**DOI:** 10.1186/s12882-015-0016-6

**Published:** 2015-02-26

**Authors:** Rajit K Basu, Ahmad Kaddourah, Tara Terrell, Theresa Mottes, Patricia Arnold, Judd Jacobs, Jennifer Andringa, Stuart L Goldstein, on behalf of the Prospective Pediatric AKI Research Group (ppAKI)

**Affiliations:** Underneath Center for Acute Care Nephrology, Cincinnati Children’s Hospital and Medical Center, Cincinnati, OH 45229 USA; Department of Pediatrics, Division of Biostatistics and Epidemiology, Cincinnati Children’s Hospital and Medical Center, University of Cincinnati, Cincinnati, OH 45229 USA; Division of Critical Care, Cincinnati Children’s Hospital and Medical Center, 3333 Burnet Avenue, ML 2005, Cincinnati, OH 45229 USA

**Keywords:** Acute kidney injury, Critical care, Pediatrics, Renal angina

## Abstract

**Background:**

Acute kidney injury (AKI) is associated with poor outcome in critically ill children. While data extracted from retrospective study of pediatric populations demonstrate a high incidence of AKI, the literature lacks focused and comprehensive multicenter studies describing AKI risk factors, epidemiology, and outcome. Additionally, very few pediatric studies have examined novel urinary biomarkers outside of the cardiopulmonary bypass population.

**Methods/Design:**

This is a prospective observational study. We anticipate collecting data on over 5000 critically ill children admitted to 31 pediatric intensive care units (PICUs) across the world during the calendar year of 2014. Data will be collected for seven days on all children older than 90 days and younger than 25 years without baseline stage 5 chronic kidney disease, chronic renal replacement therapy, and outside of 90 days of a kidney transplant or from surgical correction of congenital heart disease. Data to be collected includes demographic information, admission diagnoses and co-morbidities, and details on fluid and vasoactive resuscitation used. The renal angina index will be calculated integrating risk factors and early changes in serum creatinine and fluid overload. On days 2–7, all hemodynamic and pertinent laboratory values will be captured focusing on AKI pertinent values. Daily calculated values will include % fluid overload, fluid corrected creatinine, and KDIGO AKI stage. Urine will be captured twice daily for biomarker analysis on Days 0–3 of admission. Biomarkers to be measured include neutrophil gelatinase lipocalin (NGAL), kidney injury molecule-1 (KIM-1), liver-type fatty acid binding protein (l-FABP), and interleukin-18 (IL-18). The primary outcome to be quantified is incidence rate of severe AKI on Day 3 (Day 3 – AKI). Prediction of Day 3 – AKI by the RAI and after incorporation of biomarkers with RAI will be analyzed.

**Discussion:**

The Assessment of Worldwide Acute Kidney Injury, Renal Angina and Epidemiology (AWARE) study, creates the first prospective international pediatric all cause AKI data warehouse and biologic sample repository, providing a broad and invaluable resource for critical care nephrologists seeking to study risk factors, prediction, identification, and treatment options for a disease syndrome with high associated morbidity affecting a significant proportion of hospitalized children.

**Trial registration:**

ClinicalTrials.gov: NCT01987921

**Electronic supplementary material:**

The online version of this article (doi:10.1186/s12882-015-0016-6) contains supplementary material, which is available to authorized users.

## Background

Acute kidney injury (AKI) is associated with poor outcome in critically ill children. The reported incidence rate of AKI in children admitted to pediatric intensive care units (PICUs) range from 8% and 89% [1-6]. AKI has been associated with prolonged hospital stay, progression to chronic kidney disease, and a significantly higher relative risk of in-hospital death [7-10]. The epidemiology and outcomes of adult AKI have been validated through large, multi-center studies describing over 20,000 adult patients [11-13]. Unfortunately, the current pediatric literature lacks such extensive studies. To date, the largest study about AKI in children admitted to PICU was carried by Schneider et al. on 3396 children [1]. Despite the large population size, this was a single-center study and also did not use the pediatric RIFLE criteria to define AKI, the standard for pediatric AKI definition before the recent KDIGO consensus criteria [4,14]. Aside from a few single center studies, most knowledge of pediatric AKI is gleaned from retrospective studies with relatively small sample sizes and with diverse AKI definitions [2,4,15]. To date, there is no multicenter prospective study describing the epidemiology and outcome of pediatric AKI in PICUs.

Despite increasing awareness of the prevalence and significance of AKI, effective therapies for this condition are lacking. This, at least in part, stems from a failure to recognize AKI before a significant degree of renal damage has already occurred. The inability to diagnose AKI expeditiously follows from the fact that the currently accepted definitions of AKI rely on changes in serum creatinine (SCr) and urine output [4]. The well-recognized limitations of SCr have been previously described [16,17]. Intensive basic and translational research has targeted the discovery of biomarkers able to uncover AKI prior to elevations in serum creatinine. To date, a number of promising candidate urinary AKI biomarkers have emerged following preliminary proteomic analyses in murine models of renal ischemia [18]. Clinical studies indicate that urinary neutrophil gelatinase-associated lipocalin (NGAL), kidney injury molecule-1 (KIM-1), interleukin 18 (IL-18) and liver-type fatty acid binding protein (L-FABP) all predict AKI in children following cardiopulmonary bypass prior to changes in serum creatinine [19-21]. Widespread clinical extrapolation of these results is challenging, however, given their derivation and validation in homogenous populations free from common co-morbidities and exposed to a uniform insult on a known time scale. New adult-specific data indicate that plasma NGAL demonstrates reasonable predictive performance in heterogeneous patients, with variable comorbidities, presenting to the emergency room [22]. Neither plasma NGAL, nor the other biomarkers listed above, have demonstrated robust efficacy in children with heterogeneous illness when tested in isolation. Additionally, select early papers utilized definitions of AKI that may have contributed to the high sensitivities subsequently achieved by urinary biomarkers [23]. Such issues may underlie these urinary biomarkers' inability to predict AKI severity, identify children who would require renal replacement therapy (RRT), and predict AKI-associated death [23-27]. In response to a need of clinical predictors of AKI, we proposed the renal angina clinical model [28,29]. In this model, a composite of clinical risk factors and clinical evidence (the renal angina index (RAI)) of acute kidney injury directs biomarker testing, akin to directed assessment of troponin I only in select patients with chest pain. This model seeks a high negative predictive value (NPV) for AKI of *not fulfilling renal angina*. Further, unlike difficult to use, severity of illness scoring systems which merely score existing injury, fulfillment of renal angina aids *prediction* of severe AKI. In relatively small, retrospective studies, we have demonstrated that the RAI offers moderate discrimination for severe AKI, prediction which improves after the incorporation of biomarkers [30]. This ‘targeting’ of biomarker testing demonstrates a methodology to optimize the utility of novel diagnostics.

Given the paucity of prospective studies directly aimed at investigating pediatric AKI in critical illness, a large and diverse observational study is needed to enrich the field of pediatric critical care nephrology with current data. In this manuscript we describe the methodology of the Assessment of Worldwide AKI, Renal Angina and Epidemiology (AWARE) study. The AWARE repository will facilitate analysis of epidemiologic trends, refine risk stratification, solidify associated morbidities, identify disparities across the globe, and potentially uncover information vital to mitigating the burden of the AKI syndrome.

## Methods/Design

### Design

The design is a prospective, multi-center, observational trial of critically ill children admitted to the pediatric intensive care unit (PICU).

### Setting

The setting is 32 PICUs across 5 continents and 12 countries. Site investigators are listed in Additional file 1.

### Population

Eligible participants fulfill all inclusion and no exclusion criteria.

### Inclusion criteria

The inclusion criteria are designed to capture as many potential study patients as possible and are inclusive of most patients admitted to the PICU and cardiac intensive care unit (CICU). All inclusion criteria must be met and only patients with an ICU length of stay of at least 48 hours are included in data analysis (other patient data is kept for demographic data repository, but excluded from data analysis for renal angina or AKI associated outcome).

1. In-patient in a PICU or CICU
2. Age ≥ 90 days
3. Age < 25 years

### Exclusion criteria

1. Maintenance hemodyialysis or peritoneal dialysis.
2. Chronic kidney disease with a baseline estimated glomerular filtration rate (eGFR) of < 15 ml/min/1.73 m^2^.
3. Kidney transplant within 90 days of PICU/CICU admission.
4. Post-operative from surgical correction of cyanotic congenital heart disease within 90 days of PICU/CICU admission.
5. Uncorrected congenital heart disease (does NOT include patients with an isolated atrial or ventricular septal defect, patent ductus arteriosus, or patent foramen ovale).
6. Immediately following elective cardiac catheterization.

For exclusion criteria 4–6, patients admitted and then taken to the operating theater for surgical corrections requiring cardiopulmonary bypass are included for study.

### Urine collection

For sites that have agreed to collect urine samples, eligible patients for study will have urine collected from an indwelling urinary catheter (foley) or via clean intermittent catheterization twice daily (between 6 and 10 am and between 3 and 7 pm) within the first 48 hours of admission (and then for as many of the regularly scheduled samples as possible within the first 4 days of PICU/CICU admission). Patients are not bagged or catheterized separately/independently for the purposes of this study. Collected urine samples are kept on ice or in 4°C refrigerator until they are processed. During processing, specimens are centrifuged at 3000 RPM at 4°C for fifteen minutes. The supernatants are divided into up to nine 1-mL cryovials depending on the collected urine volume and stored at minus 80°C. The stored urine samples from all participating sites are shipped to the Center for Acute Care Nephrology/Nephrology Center of Excellence Biomarker Core Laboratory in the Division of Nephrology and Hypertension at Cincinnati Children’s Hospital Medical Center.

### Urinary biomarker sampling

Urine NGAL will be assayed using a human-specific commercially available enzyme-linked immunosorbent assay (ELISA, AntibodyShop, Grusbakken, Denmark). Urine IL-18 and L-FABP will be measured using commercially available ELISA kits (Medical & Biological Laboratories Co., Nagoya, Japan, and CMIC Co., Tokyo, Japan, respectively) per manufacturer’s instructions. Urine KIM-1 is measured by ELISA using commercially available reagents (R&D Systems, Inc., Minneapolis, Minnesota).

### Variable collection

Data collected per patient encompasses admission demographic data, daily morning hemodynamic parameters, daily laboratory values specific for kidney function, assessments of fluid balance including net fluid in and net fluid out, and use of nephrotoxins or diuretic agents. For admission epidemiology, primary ICU diagnoses are broadly divided into shock/infection/major trauma, medical cardiac, respiratory failure, post-surgical/minor trauma, central nervous system dysfunction, and pain/sedation management. Net fluid balance is divided into total fluids and urine flow rates derived per kilogram admission body weight per hour. Daily calculated values include:

1. Estimated change in creatinine clearance
  - Calculated as percent change of daily creatinine from baseline creatinine.
  - Baseline creatinine used is lowest consistent serum creatinine 90 days or more prior to admission.
  - For patients without a prior baseline, an assumed creatinine clearance of 120 ml/min/1.73 m^2^ is used [3].
2. Percent fluid overload
  - Calculated as previously described [31].
3. Fluid corrected serum creatinine
  - Calculated as previously described [32].
4. KDIGO stage AKI by creatinine
  - Based on KDIGO AKI guidelines [33].

### Renal angina index calculation

To ascertain fulfillment or absence of renal angina on the day of admission, the renal angina index will be calculated as previously described (Additional file 2) [5]. An RAI of ≥8 will be interpreted as fulfillment of renal angina.

### MediData Rave™

Data entry of the variables of interest will be performed by the investigators and clinical research coordinators at the participating sites using a web-based data base: MediData Rave™. Rave™ is a commercial system designed to capture, manage, and report clinical research data. Through this system, each participating site is assigned a unique code, as identified by the study team. If responses to the initial inclusion and exclusion criteria provided by the individual performing the data entry fulfill study criteria, the system will dynamically generate the remainder of the patient casebook, opening the “gateway” for the site to enter additional data for an enrolled patient. When eligibility is determined, the system will guide the data entry personnel at each site to enter the clinical variables of interest. All participating sites will use the same case report forms (CRFs). The electronic CRFs will be designed and monitored by the representatives in the Data Management Center (DMC) at Cincinnati Children’s Hospital Medical Center (CCHMC) based on the paper CRFs developed by the clinical team. The DMC team from the coordinating site, the Center for Acute Care Nephrology (CACN) at CCHMC, will be the only individuals that can access and extract the data for all other sites. Other sites will have access only to their enrolled subjects in Rave™. Data management and statistical analysis will be executed at CCHMC.

### Interventions

AWARE is a non-intervention observational study. Urine collection will occur only for patients that have an indwelling urinary catheter or are scheduled for clean intermittent catheterization.

### Consent

AWARE is proposed as human subjects research with a waiver of informed consent/parental permission and assent. This waiver is pursued by the following rationale:

1. The research involves no more than minimal risk to the subjects.
2. The waiver does not adversely affect the rights and welfare of the subjects.
3. The research cannot practically be carried out without the waiver or alteration. Enrolling the maximum number of PICU admissions during the study period yields the greatest and most informative amount of data. Requiring informed consent from every eligible patient causes a significant reduction in enrollment and potentially introduces selection bias into the dataset (i.e., omission of all patients from centers with limited clinical research personnel). Robust quality improvement and process improvement work in patients with acute kidney injury requires that all subjects with acute kidney injury be included in the process. Requiring informed consent leads to incomplete participation, and therefore the data gathered under an informed consent requirement reduces the reliability of the data.
4. The research on urine collection and biomarker measurements is reliably and confidentially performed with waiver of consent as long as the following caveats are applied
  - Only urine intended for discard or waste will be used.
  - Urine will be collected only from patients with an indwelling urinary drainage system and collection apparatus or scheduled for intermittent catheterization. Patients will not be bagged or catheterized separately/independently for the purposes of this study.

The sites participating in AWARE have obtained appropriate ethical board approval from their respective review consensus boards (Additional files 3 and 4). No site participating in the study is awaiting approval from an ethical board. Although some institutions have waived the need for consent, some require written, informed consent and this will be obtained as indicated to fulfill an additional inclusion criterion.

### Co-enrollment

Patients enrolled in AWARE may also be enrolled in other studies without exception. As AWARE is non-interventional, there is no overlap in the observation with other CACN or PICU/CICU origin studies.

### Primary and secondary outcomes

Our primary outcome is to report the epidemiology and associated outcomes of pediatric AKI worldwide (in over 30 PICUs, 12 different countries, 5 continents). This outcome is dependent on broad enrollment from participating centers. AKI is defined as KDIGO stage 2 or 3 AKI by creatinine and/or urine output criteria on Day 3 or later of PICU/CICU admission (Day 3 AKI).

Our secondary outcomes are to validate and refine the renal angina risk stratification model for prediction of Day 3 AKI.

1. Determine if fulfillment of renal angina on Day 0 is predictive of severe AKI on Day 3 of PICU/CICU admission across the heterogeneous patient landscape of our participating centers. The AWARE data repository will be used to validate the precision of the RAI in ruling out AKI.
2. Determine if prediction of Day 3 AKI by an RAI ≥ 8 is augmented by inclusion of urinary biomarkers alone or in combination.

Longer term outcomes to be followed include duration of mechanical ventilation, use of continuous renal replacement therapy, use of extracorporeal assist devices such as extracorporeal membrane oxygenation (ECMO) or ventricular assist devices (VADs), ICU length of stay, and mortality (Day 30 follow up).

### Sample size

The AWARE study will be the largest prospective *pediatric* AKI study describing global epidemiology, risk factors, and associated outcomes. To date, the largest prospective cohort study of AKI to date was conducted by the Beginning and Ending Supportive Therapy for the Kidney (BEST Kidney) investigators [34]. In the BEST Kidney study 18% of 29,269 patients prospectively studied after admission to 54 adult ICUs across 23 countries over 15 months developed AKI. For the AWARE study, we based our sample size estimation on the number needed to validate RAI internationally. We validated RAI in 370 PICU admissions from two different sites (average of 185 per site) in North America [5]. To be able to make RAI a universal index, we need to validate the RAI in all participating sites. Accordingly we are estimating the average number of enrolled children to be an average of 150 from each participating site with a total of approximately 5,250 children from all sites (assuming the final number of participating centers is around 35). Among the participating sites, we estimate one third to participate in urine collection, resulting in an expected approximately 1750 patients with urine biomarkers able to be measured. We are allocating each site 3 consecutive months to complete patient enrollment. Data capture can occur after the three months are complete, but no new patients are to be enrolled.

### Analysis

Analysis of data will be performed independently based on each specific aim.

1. Data for the primary objective of describing the epidemiology of AKI will be presented as a descriptive model. The prevalence on day 0 and incidence of AKI in up to 7 days of ICU admission using KDIGO classification will be calculated for each site cohort to identify the geographical “hot spots” of pediatric AKI. The data then will be pooled into a single cohort to study the outcome of AKI. The whole cohort will be stratified on Day 3 into four sub-populations with: no AKI, AKI-KDIGO Stage 1, AKI-KDIGO Stage 2, and AKI-KDIGO Stage 3. An adjusted and unadjusted survival analysis models using log rank test and cox regression models will be used to compare the mortality rates and the need of renal replacement therapy between the 4 groups.
2. The prognostic value of the renal angina index will be calculated on Day 0 for the development of Day 3 AKI. RAI will be evaluated as a diagnostic test and sensitivity, specificity, positive predictive value, negative predictive value, likelihood ratios, and receiver operating characteristics (ROCs) will be derived.
3. Individual prognostic values of each of the four urinary biomarkers al one for Day 3 AKI will be calculated. The biomarkers will be tested in combination for changes in prognostic parameters and comparisons of discrimination using net reclassification improvement (NRI) and integrated discrimination improvement (IDI) will be derived. Finally, the contribution of each biomarker to the predictive model of renal angina for Day 3 AKI will be analyzed by integrating the biomarkers alone and in combination with the RAI.

Additionally, the association of renal angina index, urinary biomarker levels with clinical outcomes, including mortality, PICU length of stay, hospital length of stay, and renal replacement therapy provision, will be assessed using Chi-square test (or Fisher’s exact test for small counts) or Pearson correlation coefficient (or non-parametric Spearman correlation coefficient) based on the nature of data. Classification and regression tree analysis will be used to determine optimal decision rules for biomarker incorporation with RAI when discriminating for Day 3 AKI. In all analyses, a p-value of <0.05 will be considered statistically significant.

### Oversight

The Center for Acute Care Nephrology at CCHMC will oversee the AWARE trial from start to finish. The central data repository through RAVE™ will be housed at CCHMC. Each site will perform screening, enrollment, consenting (when applicable), processing the urine samples (when applicable), collecting and entering data to Rave™ web browser. The research personnel in every site will be able to access the data of children enrolled from the same site. Only CCHMC research personnel will have access to the data collected for the purposes of this study from all participating sites. CCHMC will be responsible for managing and analyzing the data and testing the urine samples for urinary biomarkers. All participating sites will use the same case report form (CRF). The web-based CRFs will be designed and monitored by the Medidata Rave™ representatives in the Data Management Core at Cincinnati Children’s Hospital Medical Center (CCHMC). As the project coordinators, the Center for Acute Care Nephrology (CACN) at CCHMC will be the only site that can access the data from all other sites. Other sites will have access only to their enrolled subjects. Data management and statistical analysis will be executed at CCHMC.

The AWARE study is a featured study of the Prospective Pediatric Acute Kidney Injury Registry (www.ppaki.org). Founded in 2012, the ppAKI is an international research consortium comprised of pediatric nephrologists and intensivists striving to foster development and advances in the research of pediatric acute kidney injury.

## Discussion

The global epidemiology of pediatric acute kidney injury is unknown. What is known is that the landscape of pediatric AKI is dynamic, changing considerably in the past 30 years, from a name change (acute renal failure to acute kidney injury), to continual reclassification (RIFLE, pRIFLE, AKIN, and KDIGO), to a growing appreciation of disease severity and associated disease burden. Novel diagnostic methodologies including renal angina and urinary biomarkers are expected to advance the field of AKI diagnostics. Unfortunately, contrasted with large scale adult epidemiologic studies, robust and broad based data in pediatrics is currently unavailable. The paucity of inclusive and expansive pediatric data presents a major hurdle to the advancement of the field towards improving outcomes.

Strengths in the design of this prospective trial include the magnitude of patient enrollment (over 5,000 expected and at the time of this writing over 3,500 enrolled), the broad geographic distribution of patients (from over 5 continents and a site in South Africa still pending site institutional review board approval), and the inclusion of all ICU patients – regardless of previously documented AKI risk factors (i.e. sepsis, mechanical ventilation, cardiopulmonary bypass). The repository of data collected will inform critical care nephrologists for many years to come and allow for analysis of many epidemiologic AKI associations and refinement of renal angina for AKI prediction and classification. The capture of urine for biomarker analysis also represents the largest urine biobank repository in pediatric critical care to date for the study of AKI. Given the expected population size and data to be prospectively captured, the AWARE study will facilitate analysis of many questions surrounding AKI, both diagnostic and therapeutic. A few examples of targeted questions potentially answerable by mining the database include: a) the association of resuscitative fluids and AKI, b) delineation of predictive and associated factors between transient versus persistent AKI, c) the independent outcomes of fluid overload and oliguria in all critically ill patients, and the d) associations and outcomes of subclinical-AKI.

The design of this prospective study has limitations. We make several assumptions with regards to the expected incidence of AKI per PICU/CICU center. Differing geographic areas included in our enrollment study list and different patient demographics may have greater or less incidence of AKI risk. Many pediatric patients have no ‘baseline’ creatinine measured prior to the time of acute illness. Our assumption of a normal creatinine clearance is based on the use of this paradigm in previous study [4]. Additionally, we assume that 33% or greater of our enrollment sites will be able to capture urine for the biomarker analysis. Perhaps the greatest limitation is that this study is being independent of financial reimbursement. Study coordinators, research coordinators, and data management specialists at each site are not compensated for the exclusive purpose of this study, which has the potential to bias the enrollment strength of each center (depending on staff enthusiasm and availability). All of these limitations can also be interpreted as strengths of the study, however. Our initial results indicate that the AWARE study will assemble a broad and heterogeneous patient repository and that the pro bono work done by our coordinating sites is robust. It is our expectation that the AWARE study will serve as a model, a proof of concept that resources are in place to facilitate broad based pediatric AKI studies, for future large scale multicenter studies that are funded and sponsored by governmental and private financial support.

AKI is a significant disease syndrome affecting a large proportion of pediatric ICU patients. Existing data indicates that patients are not just dying with AKI, but *from* AKI [35]. AWARE is a first of its kind and vital study of critically ill children that will inform the pediatric critical care nephrology community of the prevalence and associations of AKI across the globe, offering new perspectives for prediction and detection of disease.

### Trial status

Recruitment is currently active at most centers, but is limited to three consecutive months from the time of initiation at each center. Patient enrollment and data capture is expected to be complete by January 1, 2015.

## Additional files

Additional file 1:
**Principal investigators at AWARE study sites.**
Additional file 2:
**The Renal Angina Index.**
Additional file 3:
**Approvals Obtained from Ethical Committees for participation in AWARE.**
Additional file 4:
**Institutional Review Board (IRB) approvals from AWARE study sites.**

## Abbreviations

AKI: Acute kidney injury
AWARE: Assessment of Worldwide AKI, Renal Angina and Epidemiology
PICU: Pediatric intensive care unit
CICU: Cardiac intensive care unit
CACN: Center for Acute Care Nephrology
CCHMC: Cincinnati Childrens Hospital Medical Center
l-FABP: Liver type fatty acid binding protein
NGAL: Neutrophil gelatinase associated lipocalin
KIM-1: Kidney injury molecule-1
IL-18: Interleukin-18
KDIGO: Kidney Diseases Improving Global Outcomes
SCr: Serum creatinine
RRT: Renal replacement therapy
RAI: Renal angina index
NPV: Negative predictive value
eGFR: Estimated glomerular filtration rate
CRF: Case report form
ECMO: Extracorporeal membrane oxygenation
NRI: Net reclassification improvement
IDI: Integrated discrimination improvement
