# Supplementary material for: Assessment of Worldwide Acute Kidney Injury, Renal Angina and Epidemiology in Critically Ill Children (AWARE): study protocol for a prospective observational study
Source: BMC Nephrol. 2015 Feb 26;16:24. doi: 10.1186/s12882-015-0016-6 (PMC4355130; doi:10.1186/s12882-015-0016-6)
Supplement: Additional file 1: — Principal investigators at AWARE study sites. [file 12882_2015_16_MOESM1_ESM.docx]

**Additional File 1**

**Investigation Site Investigators**

University of Alabama Birmingham, Children’s Hospital of Alabama David Askenazi

Children’s Hospital of Oakland Vivienne Newman

Lucille Packard Children’s Hospital of Stanford University Scott Sutherland

Yale University Olja Couloures

Vince Faustino

Nemours/Alfred l. DuPont Hospital for Children Joshua Zaritksy

Children’s Hospital Colorado Katja Gist

Children’s Healthcare of Atlanta, Emory University Matthew Paden

University of Iowa Patrick Brophy

C.S. Mott Children’s Hospital, University of Michigan David Selewski

Helen DeVos Children’s Hospital Richard Hackbarth

Children’s Mercy Hospital and Clinics Vimal Chadha

Washington University of St. Louis, Children’s Hospital Vikas Dharnidharka

Thomas Davis

University of New Mexico Craig Wong

Cohen Children’s Medical Center of New York James Schneider

Columbia University Medical Center Fangming Lin

Stony Brook Long Island Children’s Hospital Robert Woroniecki

University of Oklahoma Health Sciences Center David Myers

Monroe Carrell Children’s Hospital, Vanderbilt University Geoffrey Fleming

Texas Children’s Hospital, Baylor University Alyssa Riley

Ayse Arikan

The Sydney Children’s Hospitals Network - Randwick Stephen Alexander

Sean Kennedy

The Sydney Children’s Hospitals Network – Westmeade Dierdre Hahn

Instituto da Crianca, Sao Paolo Brazil Vera Koch

University of Edmonton Catherine Morgan

Montreal Children’s Hospital, McGill University Michael Zappitelli

Ana Peljian

Children’s and Women’s Health Center, University of British Columbia Cherry Mammen

Nanjing Children’s Hospital, Nanjing, China Songming Huang

Indraprastha Apollo Hospital, New Delhi, India Shina Menon

Department of Child Health Cipto Mangunkusumo,University of Indonesia Eka Hidayati

Department of Child Health Airlangga University

Dr. Soetomo Hospital, Surbaya, Indonesia

Risky Prasetyo

Noer Soemyarso

Ospedale Pediatrico Bambino Gesu, Rome, Italy Stephano Picca

Seoul National University Children’s Hospital, Seoul, Republic of Korea Il-Soo Ha

Hee Gyung Kang

King’s College Hospital, London, United Kingdom, Akash Deep

Institute for Mother and Child Health Care, Belgrade, Serbia Natasa Stajic

University Children’s Hospital Belgrade, Belgrade, Serbia Brankica Spasojevic
