# Supplementary material for: Assessment of Worldwide Acute Kidney Injury, Renal Angina and Epidemiology in Critically Ill Children (AWARE): study protocol for a prospective observational study
Source: BMC Nephrol. 2015 Feb 26;16:24. doi: 10.1186/s12882-015-0016-6 (PMC4355130; doi:10.1186/s12882-015-0016-6)
Supplement: Additional file 2: — The Renal Angina Index. [file 12882_2015_16_MOESM2_ESM.doc]

**Additional file 2. The Renal Angina Index**

In order to operationalize renal angina, we developed the renal angina index (RAI). RAI is derived as a composite of risk factors and clinical signs of AKI. The logic behind the equation dictates that as a patient achieves higher risk they require less “clinical sign of AKI” early on to fulfill renal angina. Similarly, if a patient has less risk but shows more overt signs of clinical AKI signs, renal angina would also be fulfilled. Per the epidemiology of AKI, the risk of AKI increases in multiplicative fashion with increased risk factors. The incidence of AKI demonstrates fold-increases (5 to 10 to 50%) for higher risk patients. This same increase is seen in the fluid overload. Risk of mortality in patients with AKI demonstrates similar fold-increases for increasing AKI severity. Thus, the creation of the renal angina index was done by a multiplicative index (instead of sum). Mathematically, this more accurately mirrored the fold-increases seen with the epidemiology of pediatric AKI. The RAI score is a composite of risk strata and clinical signs. Risk strata were given point values of 5 (very high risk), 3 (high risk), and 1 (moderate risk). Clinical signs of injury are based on changes in estimated creatinine clearance (eCrCl) or % fluid overload (% FO). The assigned point values are: 1 (ICU status and no decrease in eCrCl or <5% FO), 2 (> 5% FO or eCrCl decrease of 0-25%), 4 (>10% FO or eCrCl decrease of 25-50%), or 8 (>15% FO or eCrCl decrease of > 50%). The composite range of the RAI is therefore: 1, 2, 3, 4, 5, 6, 8, 10, 12, 20, 24, and 40.


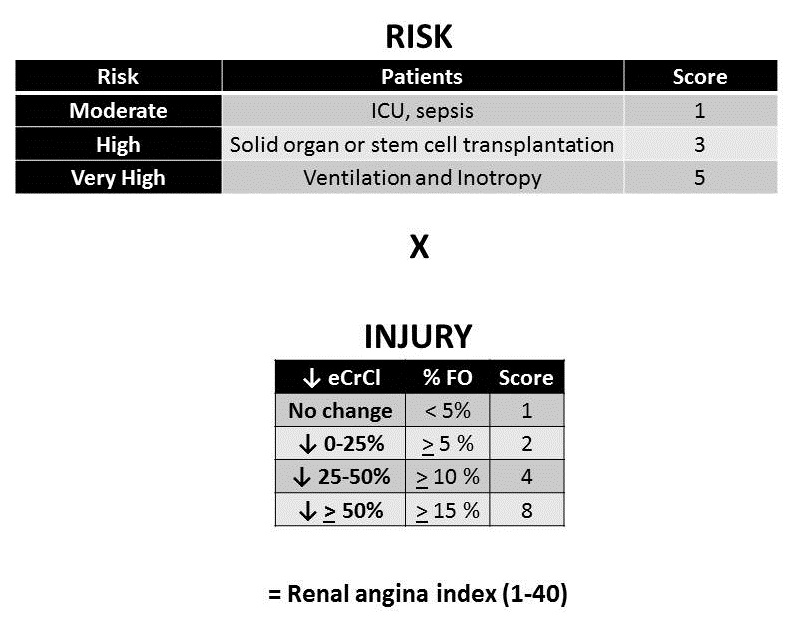


Based on the most optimal Youden’s index (C-statistic) and highest negative predictive value in derivation studies, an RAI > 8 was indicative of fulfilling renal angina.
