# Supplementary material for: Assessment of Worldwide Acute Kidney Injury, Renal Angina and Epidemiology in Critically Ill Children (AWARE): study protocol for a prospective observational study
Source: BMC Nephrol. 2015 Feb 26;16:24. doi: 10.1186/s12882-015-0016-6 (PMC4355130; doi:10.1186/s12882-015-0016-6)
Supplement: Additional file 3: — Approvals Obtained from Ethical Committees for participation in AWARE. [file 12882_2015_16_MOESM3_ESM.docx]

Additional file 3

**Approvals Obtained from Ethical Committees for participation in AWARE**

Please note, although some institutions have waived the need for consent, several institutions require written, informed patient consent and this has been taken into consideration in the inclusion criteria for AWARE (and obtained if indicated).

Sites

1. Cincinnati Children’s Hospital Medical Center
2. University of Alabama Birmingham
3. Children’s Hospital Colorado
4. Lucille Packard Children’s Hospital of Stanford University
5. Yale University
6. Nemours/Alfred l. DuPont Hospital for Children
7. Children’s Healthcare of Atlanta of Emory University
8. University of Iowa
9. C.S. Mott Children’s Hospital of the University of Michigan
10. Helen DeVos Children’s Hospital of Grand Rapids
11. Children’s Mercy Hospital and Clinics
12. Washington University of St. Louis Children’s Hospital
13. University of New Mexico
14. Cohen Children’s Medical Center of New York
15. Columbia University Medical Center
16. Stony Brook Long Island Children’s Hospital
17. Vanderbilt University
18. Texas Children’s Hospital
19. Virginia Commonwealth University
20. The Sydney Children’s Hospitals Network – Randwick
21. The Sydney Children’s Hospitals Network – Westmead
22. University of Edmonton
23. Montreal Children’s Hospital of McGill University
24. University of British Columbia and Children’s and Women’s Health Center
25. Nanjing Children’s Hospital
26. Department of Child Health Cipto Mangunkusumo of the University of Indonesia
27. Department of Child Health Airlangga University/Dr. Soetomo Hospital
28. Ospedale Pediatrico Bambino Gesu
29. Seoul National University Children’s Hospital
30. King’s College Hospital
31. Institute for Mother and Child Health Care
32. University Children’s Hospital Belgrade, Belgrade, Serbia, Brankica Spasojevic
