# Supplementary material for: Assessment of Worldwide Acute Kidney Injury, Renal Angina and Epidemiology in Critically Ill Children (AWARE): study protocol for a prospective observational study
Source: BMC Nephrol. 2015 Feb 26;16:24. doi: 10.1186/s12882-015-0016-6 (PMC4355130; doi:10.1186/s12882-015-0016-6)
Supplement: Additional file 4: — Institutional Review Board (IRB) approvals from AWARE study sites. [file 12882_2015_16_MOESM4_ESM.pdf]

Institutional Review Board - Federalwide Assurance #00002988

Cincinnati Childrens Hospital Med Ctr

Date: 10/28/2013

From: CCHMC IRB Committee

To: Principal Investigator: Stuart Goldstein
Nephrology

Re: Study ID: 2013-6501
Study Title: Assessment of Worldwide AKI, Renal Angina and Epidemiology

The above referenced protocol and all applicable additional documentation provided to the IRB were reviewed and **APPROVED** using an **EXPEDITED** review procedure in accordance with 45 CFR 46.110(b)(1)(see below) on **10/28/2013**.

This study will be due for continuing review at least 30 days before: 10/27/2014.

Study Documents

AWARE_Master_Protocol.docx

Please note the following requirements:

Consent Requirements
Per 45 CFR 46.116 the IRB has waived the requirement to obtain informed consent for all adult participants.

Parental Permission Requirements
Per 45 CFR 46.116 the IRB has waived the requirement to obtain parental permission from the parent(s) (or guardian) of all child participants. NOTE: If your research is subject to FDA regulations it is not eligible for this waiver of parental permission.

Assent Requirements

Per 45 CFR 46.116 the IRB has waived the requirement to obtain assent from all child participants. NOTE: If your research is subject to FDA regulations it is not eligible for this waiver of assent.

HIPAA Requirements
Per 45 CFR 164.512 the IRB has granted a waiver from the requirement to obtain an authorization for the use and/or disclosure of protected health information (PHI).

AMENDMENTS: The principal investigator is responsible for notifying the IRB of any changes in the protocol, participating investigators, procedures, recruitment, consent forms, FDA status, or conflicts of interest. Approval is based on the information as submitted. New procedures cannot be initiated until IRB approval has been given. If you wish to change any aspect of this study, please submit an Amendment via ePAS to the IRB, providing a justification for each requested change.

CONTINUING REVIEW: The investigator is responsible for submitting a Continuing Review via ePAS to the IRB at least 30 days prior to the expiration date listed above. Please note that study procedures may only continue into the next cycle if the IRB has reviewed and granted re-approval prior to the expiration date.

UNANTICIPATED PROBLEMS: The investigator is responsible for reporting **unanticipated problems** promptly to the IRB via ePAS according to current reporting policies.

STUDY COMPLETION: The investigator is responsible for notifying the IRB by submitting a Request to Close via ePAS when the research, including data analysis, has completed.

Please note: This approval is through the IRB only. You may be responsible for reporting to other regulatory officials (e.g. VA Research and Development Office, UC Health – University Hospital). Please check with your institution and department to ensure you have met all reporting requirements.

Statement regarding International conference on Harmonization and Good clinical Practices. The Institutional Review Board is duly constituted (fulfilling FDA requirements for diversity), has written procedures for initial and continuing review of clinical trials: prepares written minutes of convened meetings and retains records pertaining to the review and approval process; all in compliance with requirements defined in 21 CFR Parts 50, 56 and 312 Code of Federal Regulations. This institution is in compliance with the ICH GCP as adopted by FDA/DHHS.

Thank you for your cooperation during the review process.

Research Categories

3. Prospective collection of biological specimens for research purposes by noninvasive means. Examples: (a) hair and nail clippings in a nondisfiguring manner; (b) deciduous teeth at time of exfoliation or if routine patient care indicates a need for extraction; (c) permanent teeth if routine patient care indicates a need for extraction; (d) excreta and external secretions (including sweat); (e) uncannulated saliva collected either in an unstimulated fashion or stimulated by chewing gumbase or wax or by applying a dilute citric solution to the tongue; (f) placenta removed at delivery; (g) amniotic fluid obtained at the time of rupture of the membrane prior to or during labor; (h) supra- and subgingival dental plaque and calculus, provided the collection procedure is not more invasive than routine prophylactic scaling of the teeth and the process is accomplished in accordance with accepted prophylactic techniques; (i) mucosal and skin cells collected by buccal scraping or swab, skin swab, or mouth washings; (j) sputum collected after saline mist nebulization.

---

5. Research involving materials (data, documents, records, or specimens) that have been collected, or will be collected solely for nonresearch purposes (such as medical treatment or diagnosis). (NOTE: Some research in this category may be exempt from the HHS regulations for the protection of human subjects. 45 CFR 46.101(b)(4). This listing refers only to research that is not exempt.)

UNIVERZITETSKA DEČJA KLINIKA
Telefon: 011/ 20 60 652
Faks: 011/ 26 84 672
klinika@udk.bg.ac.rs
B e o g r a d
Tiršova 10

РЕПУБЛИКА СРБИЈА
УНИВЕРЗИТЕТСКА ДЕЧЈА КЛИНИКА
ПРИМЉЕНО: 11.08.2014

Орг. јед.	Број	Прилог	Вредност
017/6	261/16	0	

Na osnovu odredaba Zakona o lekovima i medicinskim sredstvima (Sl. Glasnik RS br. 30/2010. od 07.05.2010. godine), Pravilnika o uslovima i načinu kliničkog ispitivanja leka, postupku i sadržaju dokumentacije za odobrenje kliničkog ispitivanja leka (Sl. Glasnik RS br. 19/07), Poslovnika o radu Etičkog odbora Univerzitetske dečje klinike, postupajući u skladu sa Načelima dobre kliničke prakse (GCP), Etički odbor Univerzitetske dečje klinike u sastavu:

Asist. dr Vera Zdravković, pedijatar, predsednik
Prof. dr Dragana Janić, pedijatar, zamenik predsednika
Doc. Dr Dragana Bogićević, pedijatar, član
Dr Ivan Rakić, dečji hirurg, član
Doc. dr Vojislav Parezanović, pedijatar, član
Prof. dr Milica Bajčetić, klinički farmakolog, član
Dipl. pravnik Branka Krunić, pravnik u Kliničkom centru Srbije, član

odlučujući o zahtevu dr Brankice Spasojević, na svojoj sednici održanoj dana 01.08.2014. godine, doneo je jednoglasno sledeću

ODLUKU

ODOBRAVA SE sprovođenje istraživanja pod nazivom: "*„Assesment of Worldwide Acute Kidney Injury, Renal Angina and Epidemiology" (The AWARE Study)*".

O b r a z l o ž e n j e

Na svojoj sednici održanoj dana 01.08.2014. godine, Etički odbor je razmatrao zahtev za odobrenje istraživanja pod nazivom: "*„Assesment of Worldwide Acute Kidney Injury, Renal Angina and Epidemiology" (The AWARE Study)*".

Razmatrajući celokupnu priloženu dokumentaciju, Etički odbor je, shodno ovlašćenjima iz odredbe člana 36 Pravilnika o uslovima i načinu kliničkog ispitivanja leka, postupku i sadržaju dokumentacije za odobrenje kliničkog ispitivanja leka („Službeni glasnik RS" 102/06), konstatovao da je ista u skladu sa načelima i smernicama dobre kliničke prakse (GCP), te je saglasno svemu navedenom doneo odluku kao u izreci.

UNIVERZITETSKA DEČJA KLINIKA
ZA ETIČKI ODBOR

PREDSEDNIK

Asistent dr Vera Zdravković

Dostaviti

-Službi za opšte i pravne poslove
- podnosiocu zahtev
- a/a

Retrospective Collection

University of Colorado
Anschutz Medical Campus

Colorado Multiple Institutional Review Board, CB F490
University of Colorado, Anschutz Medical Campus
13001 E. 17th Place, Building 500, Room N3214
Aurora, Colorado 80045

303.724.1055 [Phone]
303.724.0990 [Fax]
COMIRB Home Page [Web]
comirb@ucdenver.edu [E-Mail]
FWA00005070 [FWA]

University of Colorado Hospital
Denver Health Medical Center
Veteran's Administration Medical Center
The Children's Hospital
University of Colorado Denver
Colorado Prevention Center

Certificate of Approval

25-Jul-2014

**Investigator:**	Katja Gist
**Sponsor(s):**	
**Subject:**	COMIRB Protocol 14-1269 Initial Application
**Effective Date:**	24-Jul-2014
**Expiration Date:**	23-Jul-2015
**Expedited Category:**	5
**Title:**	Assessment of Worldwide Acute Kidney Injury (AKI), Renal Angina and Epidemiology (The AWARE Study)

Submission ID: APP001-2
Description:

Expedited Initial Review

Response to Minor Modifications

All COMIRB Approved Investigators must comply with the following:

- For the duration of your protocol, any change in the experimental design/consent and/or assent form must be approved by the COMIRB before implementation of the changes.
- Use only a copy of the COMIRB signed and dated Consent and/or Assent Form. The investigator bears the responsibility for obtaining from all subjects "Informed Consent" as approved by the COMIRB. The COMIRB REQUIRES that the subject be given a copy of the consent and/or assent form. Consent and/or assent forms must include the name and telephone number of the investigator.
- Provide non-English speaking subjects with a certified translation of the approved Consent and/or Assent Form in the subject's first language.
- The investigator also bears the responsibility for informing the COMIRB immediately of any Unanticipated Problems that are unexpected and related to the study in accordance with COMIRB Policy and Procedures.
- Obtain COMIRB approval for all advertisements, questionnaires and surveys before use.
- Federal regulations require a Continuing Review to renew approval of this project within a 12-month period from the last approval date unless otherwise indicated in the review cycle listed below. If you have a restricted/high risk protocol, specific details will be outlined in this letter. Non-compliance with Continuing Review will result in the termination of this study.

You will be sent a Continuing Review reminder 75 days prior to the expiration date. Any questions regarding this COMIRB action can be referred to the Coordinator at 303-724-1055 or UCHSC Box F-490.

Review Comments:

This electronic approval includes:

-Response Form dated 7/22/14
-Application and Attachments A,F,H,Attachment M: Full Waiver of Consent: Determined to meet criteria for full waiver of consent for entire study, Attachment O: Full Waiver of HIPAA Authorization: Determined to meet criteria for full waiver of HIPAA authorization for entire study, P and S dated 7/22/14
-Personnel C
-COMIRB Protocol dated 7/22/14
-Cincinnati Children's Hospital Medical Center Protocol dated 1/15/14
-Case Report Form dated 1/27/14
-Manual of Operating Procedures dated 3/24/14

Affiliated Sites:
-Children's Hospital Colorado

Non-affiliates Sites: 30

NOTE TO PI:
Please submit an amendment with a copy of the completed Data Use Agreement to COMIRB when it is available.

Please note that COMIRB will no longer be E-mailing approved documents. Stamped, approved documents can be retrieved in the eRA (InfoEd) system. Please click here to access instructions on finding these approved documents.

Sincerely,

UCD Panel C

Please provide your feedback on IRB processes and support

University of Colorado Anschutz Medical Campus

Colorado Multiple Institutional Review Board, CB F490
University of Colorado, Anschutz Medical Campus
13001 E. 17th Place, Building 500, Room N3214
Aurora, Colorado 80045

303.724.1055 [Phone]
303.724.0990 [Fax]
COMIRB Home Page [Web]
comirb@ucdenver.edu [E-Mail]
FWA00005070 [FWA]

University of Colorado Hospital
Denver Health Medical Center
Veteran's Administration Medical Center
The Children's Hospital
University of Colorado Denver
Colorado Prevention Center

Certificate of HIPAA Compliance

25-Jul-2014

**Investigator:**	Katja Gist
**Sponsor(s):**	
**Subject:**	COMIRB Protocol 14-1269 HIPAA
**Effective Date:**	24-Jul-2014
**Title:**	Assessment of Worldwide Acute Kidney Injury (AKI), Renal Angina and Epidemiology (The AWARE Study)

Based upon information submitted to COMIRB, this protocol meets the requirements for HIPAA Compliance in its use of:

Submission ID: HIP001-1
Attachment O: Request for HIPAA waiver

Review Comments:

Full Waiver of HIPAA Authorization approved: Determined to meet criteria for full waiver of HIPAA authorization for entire study.

Please note that COMIRB will no longer be E-mailing approved documents. Stamped, approved documents can be retrieved in the eRA (InfoEd) system. Please click here to access instructions on finding these approved documents.

Sincerely,

UCD Panel C

Stony Brook Research

Stony Brook University Institutional Review Board (IRB)

DATE:	June 3, 2014
TO:	Robert Woroniecki
FROM:	Stony Brook University IRB (CORIHS B)
SUBMISSION TYPE:	New Project
STUDY TITLE:	[549757-3] Assessment of Worldwide Acute Kidney Injury (AKI), Renal Angina and Epidemiology (The AWARE Study)
CORIHS#:	2014-2526-F
ACTION:	APPROVED
MEETING DATE (IF FULL REVIEW):	
SUBMISSION APPROVAL DATE:	June 03, 2014
PROJECT EXPIRATION DATE:	June 02, 2015
REVIEW TYPE:	Expedited
EXPEDITED REVIEW CATEGORY:	#3,5

Thank you for your submission of New Project materials for this research study. Stony Brook University IRB (CORIHS B) (FWA #00000125) has APPROVED your submission.

All research must be conducted in accordance with this approved submission. Any modifications to the study as approved must be reviewed and approved by CORIHS prior to initiation.

If this activity has components that require approval from additional compliance committees (e.g., IACUC, IRB, IBC, SCRO, COI) it is your responsibility to not commence with the study until these approvals have been secured as well.

Please note:

- Consent forms signed by subjects in this study must be kept by the investigator for 6 (six) years from study termination, or indefinitely (if so indicated in the consent form).
- Inclusion of minors in this study is acceptable in accordance with 45 CFR 46.404
- Parental permission and minor assent is obtained in accordance with 45 CFR 46.408. Minor assent is also obtained in accordance with SBU Assent Policy, Category 1.
- Approval includes: Protocol version uploaded 4/23/14.

FOR NEW STUDIES ONLY:
If your study involves University Hospital patients, facilities, personnel and/or services, your study must not commence until you receive documented approval from: Joseph Laver (all studies), Rhona Vainder (Chernoff) or Regina Rigoroso (all studies), **Jay Bock** (if your study involves

Generated on IRBNet

Pathology/Laboratory Services), **'The Research Pharmacy' Group** (if your study involves the Pharmacy), **Mark Schweitzer** (if your study requires Radiology services), and **Stephanie Musso** (if identifiable subject health information will be electronically transmitted outside of SBU).

You are reminded that you must apply for, undergo review, and be granted continued approval for this study before June 02, 2015 in order to be able to conduct your study in an uninterrupted manner. If you do not receive approval before this date, you must cease and desist all research involving human subjects, their tissue and their data until such time as approval is granted.

Where obtaining informed consent/permission/assent is required as a condition of approval, be sure to assess subject capacity in every case, and continue to monitor the subject's willingness to be in the study throughout his/her duration of participation. Only use current CORIHS-stamped forms in the consent process. Each subject must receive a copy of his/her signed consent/permission/assent document.

Unanticipated problems (including serious adverse events) must be reported to this office in accordance with SBU Policy at: https://web.stonybrook.edu/research/humans-sop/Shared%20Documents/Section08.aspx.

Any complaints or issues of non-compliance must be immediately reported to this office. If you have any questions or comments about this correspondence, please contact:

Office of Research Compliance
Division of Human Subject Protections
Stony Brook University
Stony Brook, NY 11794-3368.
Phone: 631-632-9036
Fax: 631-632-9839

Please include your study title and CORIHS # in all correspondence with this office.

We are interested in receiving feedback regarding your experience with the Office of Research Compliance, SBU's IRBs (CORIHS), or any other aspect of our Human Research Protection Program. Please feel free to e-mail Judy Matuk, Assistant Vice President for Research Compliance, at judy.matuk@stonybrook.edu, or if you'd like to submit feedback anonymously, you may do so at http://www.tellmyirb.com, choose the option 'Report a Problem', type in 'Stony Brook' as the site, click the radio button on the following screen, and then provide feedback!

Generated on IRBNet

Approval Form

Date:	July 9, 2014
Study ID:	Pro00049215
Principal Investigator:	Sean Bagshaw
Study Title:	Assessment of Worldwide Acute Kidney Injury (AKI), Renal Angina and Epidemiology (The AWARE Study)
Approval Expiry Date:	July 8, 2015

Thank you for submitting the above study to the Health Research Ethics Board - Health Panel . Your application, including revisions received July 9, 2014, has been reviewed and approved on behalf of the committee.

The Health Research Ethics Board assessed all matters required by section 50(1)(a) of the Health Information Act. It has been determined that the research described in the ethics application is a retrospective chart review for which subject consent for access to personally identifiable health information would not be reasonable, feasible or practical. Subject consent therefore is not required for access to personally identifiable health information described in the ethics application.

In order to comply with the Health Information Act, a copy of the approval form is being sent to the Office of the Information and Privacy Commissioner.

A renewal report must be submitted next year prior to the expiry of this approval if your study still requires ethics approval. If you do not renew on or before the renewal expiry date (July 8, 2015), you will have to re-submit an ethics application.

Approval by the Health Research Ethics Board does not encompass authorization to access the patients, staff or resources of Alberta Health Services or other local health care institutions for the purposes of the research. Enquiries regarding Alberta Health approvals should be directed to (780) 407-6041. Enquiries regarding Covenant Health approvals should be directed to (780) 735-2274.

Sincerely,

Anthony S. Joyce, Ph.D.
Chair, Health Research Ethics Board - Health Panel

Note: This correspondence includes an electronic signature (validation and approval via an online system).

Retrospective

INSTITUT ZA ZDRAVSTVENU ZAŠTITU
MAJKE I DETETA SRBIJE
"Dr VUKAN ČUPIĆ" NOVI BEOGRAD
Ul. Radoja Dakića 8, tel. 011/3108-129
Del.br. 8/21
Datum: 09 JUL 2014

Etički odbor Instituta za zdravstvenu zaštitu majke i deteta Srbije „Dr Vukan Čupić"

Etički odbor Instituta za zdravstvenu zaštitu majke i deteta je na svojoj sednici 08.07.2014. godine razmatrao zahtev za odobrenje sprovođenja nekomercijalnog kliničkog ispitivanja pod nazivom: "Assessment of Worldwide Acute Kidney Injury (AKI), Renal Angina and Epidemiology " (The AWARE Study). Glavni istraživač u Institutu je dr Nataša Stajić.

Na sednici su bili prisutni sledeći članovi Etičkog odbora:

- **Dr sc. med. Katarina Sedlecky**, specijalista ginekologije i akušerstva, zaposlena u Institutu za zdravstvenu zaštitu majke i deteta Srbije „Dr Vukan Čupić", predsednik Etičkog odbora,
- **Prim. dr Ljubica Nikolić, mr sc. med**, specijalista pedijatrije, zaposlena u Institutu za zdravstvenu zaštitu majke i deteta Srbije „Dr Vukan Čupić", potpredsednik Etičkog odbora
- **Prim. dr sc. med. Žarko Puzigaća**, specijalista ginekologije i akušerstva, zaposlen u Institutu za zdravstvenu zaštitu majke i deteta Srbije „Dr Vukan Čupić", član Etičkog odbora
- **Dr Dragana Lekić, mr sc. med**, specijalista anestezije sa reanimacijom, zaposlena u Institutu za zdravstvenu zaštitu majke i deteta Srbije „Dr Vukan Čupić", član Etičkog odbora
- **Doc. dr Vladislav Vukomanović**, specijalista pedijatrije, zaposlen na Medicinskom fakultetu Univerziteta u Beogradu i u Institutu za zdravstvenu zaštitu majke i deteta Srbije „Dr Vukan Čupić", član Etičkog odbora.
- **Hajrija Mujović – Zornić PhD, diplomirani pravnik,** zaposlena u Institutu društvenih nauka, Centar za pravna istraživanja, član Etičkog odbora
- **Jasmina Roskić, diplomirani pravnik**, zaposlena u Ministarstvu ekonomije i regionalnog razvoja, član Etičkog odbora

Etički odbor Instituta za zdravstvenu zaštitu majke i deteta je osnovan i radi u skladu sa principima dobre kliničke prakse.

Članovi Etičkog odbora su dali saglasnost da se u Institutu sprovodi nekomercijalno kliničko ispitivanje pod nazivom: "Assessment of Worldwide Acute Kidney Injury (AKI), Renal Angina and Epidemiology " (The AWARE Study). Glavni istraživač u Institutu je dr Nataša Stajić.

Novi Beograd,

09.07.2014.god.

Predsednik Etičkog odbora

Dr sc. med. Katarina Sedlecky

Hôpital de Montréal pour enfants
Centre universitaire de santé McGill

Montreal Children's Hospital
McGill University Health Centre

June 19, 2014

Dr. M. Zappitelli
Pediatric Nephrology
MUHC – Montreal Children's Hospital
Room E-213

Re: # 14-086-PED **Assessment of Worldwide Acute Kidney Injury (AKI), Renal Angina & Epidemiology**

Dear Dr. Zappitelli,

The Pediatric (PED) Research Ethics Board (REB) received an Application to Conduct Health Record Research for the study identified above. We are pleased to inform you the study complies with ethical guidelines for conduct at the McGill University Health Centre (MUHC). The study was assigned MUHC Study Code **14-086-PED** as the required MUHC reference.

Final ethics approval for the study including the Research Protocol (version 6 Sept. 2013) was provided via expedited review of the Chair on June 19, 2014. This decision will be reported to PED REB at the next meeting, and will be entered accordingly into the minutes.

All research involving human subjects requires ongoing review and the current ethics approval for the study will be in effect **until June 18, 2015.** Prior to the expiration of ethics approval, it is the Principal Investigator's responsibility to submit an "Application for Continuing Review" or a Study Completion Report to the REB.

The PED REB is a registered MUHC REB working under the published guidelines of the Tri-Council Policy Statement 2, in compliance with the "Plan d'action ministériel en éthique de la recherche et en intégrité scientifique" (MSSS, 1998) and the Food and Drugs Act (7 June, 2001), acting in conformity with standards set forth in the (US) Code of Federal Regulations governing human subjects research, and functioning in a manner consistent with internationally accepted principles of good clinical practice. At the MUHC, sponsored research activities that require US federal assurance are conducted under Federal Wide Assurance (FWA) 00000840.

You must report to the REB promptly without delay should a modification to the study be proposed, or if an unanticipated problem occurs. Regulations do not permit initiating a proposed study modification prior to REB approval for the study amendment; except where urgent action is required to eliminate an apparent immediate hazard to a study subject or other person.

It is important to note you may initiate the study once all MUHC reviews have been completed and all decisions are favorable. At that time you will receive MUHC Authorization to conduct the study issued by the Research Institute of the MUHC.

We trust this will meet with your complete satisfaction.

2300 rue Tupper, Montréal QC CANADA H3H 1P3 Téléphone : 514 412-4400
lechildren.com • thechildren.com • cusm.ca • muhc.ca

Sincerely,

Jane McDonald, MD, FRCPC
Chairperson
Pediatric Research Ethics Board

Cc: S. Lamarche

Nemours

Nemours Office of Human Subjects Protection
10140 Centurion Parkway North
Jacksonville, FL 32256
Phone: 904-697-4023 Fax: 904-697-4024

MEMORANDUM

DATE:	April 30, 2014
TO:	Joshua Zaritsky, MD
FROM:	Nemours IRB 2
STUDY TITLE:	[596181-2] AWARE - Assessment of Worldwide Acute Kidney Injury (AKI), Renal Angina and Epidemiology
IRB #:	596181
SUBMISSION TYPE:	Response/Follow-Up: New Study
ACTION:	APPROVED
APPROVAL DATE:	April 15, 2014
EXPIRATION DATE:	April 14, 2015

Thank you for your submission of Response/Follow-Up materials for this research study. Your initial submission received expedited review and met all DHHS criteria for approval. The approval was contingent on the response to minor stipulations. Your response has received expedited review and is accepted. The above-referenced research study is approved per expedited category 4.

The IRB requires that a copy of the participant brochure: "Becoming A Research Volunteer" will be given to every individual enrolled in a research study. The PDF file for this document has been attached to this study as a Board Document.

The IRB has determined that:

- This is "Research not involving greater than minimal risk per 45CFR46.404".
- Informed Consent or Parental Permission is required prior to initiation of any research procedures using only the most current IRB approved form(s) posted as a Board Document in IRBNet. All protocol documents, including Board approved documents are found in the "Study Designer" for each study in IRBNet.
- The permission of one parent is sufficient. A person who is not a parent may not give permission without prior IRB review and approval.
- Assent of minors is required prior to initiation of any research procedures, using only the most current assent form(s) posted as a Board Document in IRBNet.
- The research does not meet the criteria for including a copy of the PPF/ICF and research data in the Nemours' medical record.
- To continue, the research requires IRB review and approval on an annual basis. April 14, 2015 is the last day that research may be conducted. The Principal Investigator is responsible for the timely submission of the continuing review application. Please post this date on your research calendar.

Reviewed/approved documents in this submission:

- Consent Form - revised ICF clean version (UPDATED: 04/24/2014)

Generated on IRBNet

- Cover Sheet - Response to IRB (UPDATED: 04/24/2014)
- Other - CRRC determination (UPDATED: 04/24/2014)
- Parental Permission Form - revised ppf clean version (UPDATED: 04/24/2014)
- Application Form - Initial IRB application.docx (UPDATED: 04/10/2014)
- Child Assent - 12-17 assent (UPDATED: 04/9/2014)
- Child Assent - 7-11 assent (UPDATED: 04/9/2014)
- Data Collection - AWARE_Case Report Forms_Version 4.pdf (UPDATED: 04/10/2014)
- Investigator Agreement - Slowik (UPDATED: 04/9/2014)
- Investigator Agreement - LaRosa (UPDATED: 04/9/2014)
- Investigator Agreement - koletty (UPDATED: 04/9/2014)
- Investigator Agreement - zaritsky (UPDATED: 04/9/2014)
- Other - IBC waiver (UPDATED: 04/9/2014)
- Protocol - AWARE_Master Protocol_Version 2.pdf (UPDATED: 04/10/2014)

Investigator Agreement: As the PI, you have agreed to assure that this research is conducted in compliance with Nemours policy and all applicable federal regulations and ICH standards, including, but not inclusive of:

- All research must be conducted in accordance with this approved submission. Any revision to approved materials must be approved by the IRB prior to initiation.
- Remember that informed consent/parental permission is a process beginning with a description of the study and insurance of participant understanding followed by a signed consent form. Informed consent must continue throughout the study via a dialogue between the researcher and research participant. Federal regulations require each participant receive a copy of the signed consent document.
- All serious and unexpected adverse events and unanticipated problems affecting participants must be reported promptly to the IRB according to NOHSP policy.
- All non-compliance issues or complaints regarding this study must be reported to the Director, NOHSP.
- All research records must be retained for a minimum of three years.
- A Closure Report must be submitted to the IRB when this protocol is completed.

If you have any questions, please contact Laurie Ward at Nemours Children's Clinic, 807 Children's Way, Jacksonville, FL 32207, (904) 697-3415 or lward@nemours.org. Please include your study title and reference number in all correspondence with this office.

Researcher Correspondence Disposition for Protocol

IRB Protocol IRB-AAAN0450
Current Status: Approved

Message Text from IRB Office:

On April 14, 2014, the above-mentioned study was reviewed and approved by the Chair or Designee of Columbia University Medical Center Institutional Review Board (IRB) Exp. It met the regulatory guidelines for expedited review, category 5. You may now begin human research for this study.

The requirement to obtain informed consent from the subjects has been waived by the IRB in accordance with 45 C.F.R. § 46.116(d).

The following HIPAA document(s) were approved for use for this study: HIP-AAAJ7051 and HIP-AAAJ7210

Any proposed changes in the protocol must be immediately submitted to the IRB for review and approval prior to implementation, unless such a change is necessary to avoid immediate harm to the participants. Additionally, any unanticipated problems that involve risks to subjects must be reported to the IRB in accordance with the CUMC Unanticipated Problems: Reporting to the IRB of Unanticipated Problems Involving Risks policy, dated January 24, 2008. All submissions for modifications and unanticipated problems must be submitted through RASCAL.

Renewal applications should be submitted 60 days before the expiration date of this study through RASCAL. Failure to obtain renewal of your study prior to the expiration date will require discontinuance of all research activities for this study, including enrollment of new subjects. You must inform the IRB in writing when your study has been completed.

If you have any questions regarding this approval, please contact Susie Kim at (212) 342-3058 or sjk2142@columbia.edu.

Columbia University appreciates your commitment towards the ethical conduct of human research.

Last 10 correspondence messages

From	To	Notification Date	View Text
IRB Office	Researcher	04/22/2014 at 10:53:49	
IRB Office	Researcher	04/08/2014 at 13:34:07	
IRB Office	Researcher	01/08/2014 at 11:17:29	

View History

Edit and Respond to IRB Office (will remove from queue)

Review All Messages

Remove from queue

Exit

North Shore LIJ
North Shore-Long Island Jewish Health System

Institutional Review Board
FWA #00002505
Office of the Human Research Protection Program
3333 New Hyde Park Road, Suite 317
New Hyde Park, NY 11042
Phone: 516-719-3100 or 516-321-2100
Fax: 516-321-2125

To: James Schneider, MD
Pediatric Critical Care
Cohen Children's Medical Center
269-01 76 Avenue
New Hyde Park, NY 11040

From: Victor Fornari, MD
Chair, Institutional Review Board

Victor Fornari, MD

Date: Monday, March 31, 2014

RE:
IRB #: 14-132A
Protocol Title: Assessment of Worldwide Acute Kidney Injury (AKI) Renal Angina and Epidemiology (AWARE)
Expiration Date: 3/30/2015
Pediatric Risk Category: **Not greater than minimal risk.**

Dear Dr. Schneider:

The above referenced project meets the criteria outlined in 45 CFR 46.110 and 21 CFR 56.110 for EXPEDITED REVIEW and has been approved. The following category(ies) apply(ies) to the project:

45 CFR 46.110 (5): **Research involving materials (data, documents, records, or specimens) that have been collected, or will be collected solely for nonresearch purposes (such as medical treatment or diagnosis).**

Pre Meeting Action: **Expedited Approval**. Approval of this project includes:

1. Protocol (version 2, 1/16/14)
2. This study has been granted a waiver of informed consent and HIPAA authorization
3. The following people are approved as personnel on the study: James Schneider, Christine Sethna, Aaron Kessel, Dawn McLaughlin, Carina Iezzi, and Daniel Jun

The Institutional Review Board - Committee will be notified of this action at its meeting on 4/2/2014. This study has not been approved for the inclusion of pregnant women or prisoners. If you would like to include these populations, please notify the IRB for further instruction.

The IRB approval expiration date is listed above. As a courtesy, approximately 60 to 90 days prior to expiration of this approval, the Office of the IRB will send an e-mail reminding you to apply for continuing review. Failure to receive renewal notification does not relieve you of your responsibility to provide the Progress Report to the IRB in time for the request to be processed and approved prior to your expiration date. **It is your responsibility to apply for continuing review and receive continuing approval for the duration of the study.** Lapses in approval should be avoided to protect the safety and welfare of enrolled subjects.

Subject recruitment methods are appropriate, there is equitable selection of subjects, and there are provisions to protect and maintain the confidentiality of data and research participants.

Investigators are reminded that research must be conducted in accordance with all applicable Department of Health and Human Services regulations 45 CFR 46, Food and Drug Administration regulations 21CFR 50, 21CFR 56, 21 CFR 312, 21 CFR 812, and the Health Insurance Portability and Accountability Act (HIPAA).

All studies are subject to audits by the Office of Research Compliance and/or Institutional Review Board to confirm adherence to institutional, state, and federal regulations governing research.

NOTE: This approval is subject to recall if at any time the conditions and requirements as specified in the IRB Policies and Procedures are not followed (see next page and web site: http://www.northshorelij.com/body.cfm?ID=2804)

Internal #: 26123

NOTE: All IRB Policies and Procedures must be followed, including the following:

1. Using only IRB-approved consent forms, questionnaires, letters, advertisements, etc. in your research.
2. Submitting any modifications made to the study for IRB review prior to the initiation of changes except when necessary, to eliminate apparent, immediate hazards to the subject.
3. Reporting unanticipated problems involving risk to subjects or others.
4. Prior to implementation, any changes made to studies utilizing TAP must have COPP, as well as IRB approval.

IMPORTANT REMINDER: The International Committee of Medical Journal Editors (ICMJE) requires registration of clinical research studies meeting specific guidelines prior to publication. Please see ICMJE requirements for registration of clinical trials at http://www.icmje.org. *Our organization account is in the name of the North Shore-Long Island Jewish Health System.* To register your trial: http://prsinfo.clinicaltrials.gov . **You must register your trial PRIOR TO ENROLLING SUBJECTS.**

Children's Mercy HOSPITALS & CLINICS | Kansas City

Children's Mercy Hospital Pediatric Institutional Review Board

NOTIFICATION OF INITIAL APPROVAL

3/5/2014 9:36 AM

From:	Office of Research Integrity
To:	Vimal Chadha , Principal Investigator
CC:	Janelle Jennings
IRB#:	14020061
Study Title:	Assessment of Worldwide Acute Kidney Injury (AKI), Renal Angina and Epidemiology
Funding:	Nephrology
Protocol Name/#:	The AWARE Study

Dear Dr. Chadha,

On 3/5/2014, the CMH Pediatric IRB reviewed the above-titled new study by **expedited review** under 45 CFR 46.110 category 5. The IRB approved the submission from **3/5/2014** to **3/4/2015**.

The IRB approved the MARS application as of 3/5/2014 which included the following study documents:

Name	Modified	Version
The Aware Study | History	2/11/2014 9:44 AM	0.01
Case Report Form | History	3/4/2014 8:50 AM	0.01
Master List | History	2/25/2014 1:54 PM	0.01

Approved Permission/Assent/Consent Process(es):

Waiver of parental permission (cannot be applied to FDA regulated research)
Waiver of child assent
Waiver of consent for adult subjects (cannot be applied to FDA regulated research)

Approved HIPAA Authorization Process(es):

Waiver of HIPAA Authorization

Reminder of Principal Investigator Responsibilities:

- You are required to submit a continuing review report within MARS *30 days prior to your expiration date*. If continuing review approval is not granted before the expiration date of **3/4/2015** approval of this protocol expires on that date.
- Notify the IRB immediately upon termination of the project and/or departure of the Principal Investigator from the institution or project.
- **Report any changes or deviations in the protocol to the IRB prior to implementation.**
- Report any unexpected significant adverse events or problems related to your study promptly to the IRB.
- Maintain copies of all pertinent information related to research activities, including copies of all signed informed consent agreements obtained from participants (if applicable).

Sincerely,

Doug Swanson, M.D.
Co-Chair, CMH Pediatric Institutional Review Board
Dane Sommer, D.Min.
Co-Chair, CMH Pediatric Institutional Review Board
Rebecca A. Ballard, J.D., M.A.
Director, Office of Research Integrity

Office of Research Integrity
Children's Mercy Hospitals & Clinics
Phone: (816) 701-4358

Fax: (816) 701-4357

Email: MARSAdmin@cmh.edu

UNM HEALTH SCIENCES CENTER

Human Research Review Committee
Human Research Protections Office

February 8, 2014

Craig Wong
cwong@salud.unm.edu

Dear Dr. Wong:

On 2/8/2014, the HRRC reviewed the modifications submitted for the following:

Type of Review:	Initial Study
Title of Study:	Assessment of Worldwide Acute Kidney Injury (AKI), Renal Angina and Epidemiology
Investigator:	Craig Wong
Study ID:	13-596
Funding:	None
Grant ID:	None
IND, IDE, or HDE:	None
Submission Summary:	New study
Documents Reviewed:	• AWARE_Master Protocol_Version 2<br>• Local Protocol Addendum_1_22_14
Review Category:	Full Committee
Determinations/Waivers:	Waiver of HIPAA authorization; Waiver/alteration of the consent process; Children

The HRRC approved the study from 02/08/2014 to 12/16/2014 inclusive. Forty-five days prior to 12/16/2014, please submit a continuing review or closure request with required explanations. You can submit a continuing review or closure by navigating to the active study in Click and clicking Create Modification / CR.

If continuing review approval is not granted before the expiration date of 12/16/2014, approval of this study expires on that date.

In conducting this study, you are required to follow the Investigator Manual (HRP-103), which can be found by navigating to the IRB Library.

Sincerely,

Mark Holdsworth, PharmD
Executive Chair

Washington University in St. Louis

Human Research Protection Office

Barnes Jewish Hospital
St. Louis Children's Hospital
Washington University

IRB ID #: 201312051

To: Vikas Dharnidharka

From: The Washington University in St. Louis Institutional Review Board,
WUSTL DHHS Federalwide Assurance #FWA00002284
BJH DHHS Federalwide Assurance #FWA00002281
SLCH DHHS Federalwide Assurance #FWA00002282

Re: Assessment of Worldwide Acute Kidney Injury (AKI) Renal Angina and Epidemiology (The AWARE Study)

Protocol Number:
Protocol Version:
Protocol Date:
Amendment Number/Date(s):

Approval Date: 01/28/14 (Full Board)

This project has been granted a full waiver of HIPAA Authorization based on the documentation provided by the researcher in the myIRB application Section IV and the assurance document signed by the Principal Investigator.

This full waiver of authorization satisfies the following criteria:
(1) The use or disclosure of the requested information involves no more than a minimal risk to the privacy of individuals based on, at least, the presence of the following elements:
(a) An adequate plan to protect the identifiers from improper use and disclosure
(b) An adequate plan to destroy the identifiers at the earliest opportunity consistent with conduct of the research, unless there is a health or research justification for retaining the identifiers or such retention is otherwise required by law; and
(c) Adequate written assurances that the requested information will not be reused or disclosed to any other person or entity, except as required by law, for authorized oversight of the research study, or for other research for which the use or disclosure of the requested information would be permitted by the Privacy Rule;
(2) The research could not practicably be conducted without the waiver or alteration; and
(3) The research could not practicably be conducted without access to and use of the requested information.

This approval has been electronically signed by IRB Chair or Chair Designee:
Rick Chandel, High School
01/30/14 1133

Washington University in St. Louis

Human Research Protection Office

Barnes Jewish Hospital
St. Louis Children's Hospital
Washington University

IRB ID #: 201312051

To: Vikas Dharnidharka

From: The Washington University in St. Louis Institutional Review Board,
WUSTL DHHS Federalwide Assurance #FWA00002284
BJH DHHS Federalwide Assurance #FWA00002281
SLCH DHHS Federalwide Assurance #FWA00002282

Re: Assessment of Worldwide Acute Kidney Injury (AKI) Renal Angina and Epidemiology (The AWARE Study)

Approval Date: 01/28/14

Next IRB Approval Due Before: 01/27/15

Type of Application:

☒ New Project
☐ Continuing Review
☐ Modification

Type of Application Review:

☒ Full Board:
Meeting Date: 01/28/14
☐ Expedited
☐ Exempt
☐ Facilitated

Approved for Populations:

☒ Children
- ☐ Signature from one parent
- ☐ Signature from two parents

☐ Prisoners
☐ Pregnant Women, Fetuses, Neonates
☐ Wards of State
☐ Decisionally Impaired

660 South Euclid Ave., Campus Box 8089, St. Louis, MO 63110 Phone: (314) 633-7400 FAX: (314) 367-3041

MATERIALS APPROVED

This approval has been electronically signed by IRB Chair or Chair Designee:
Rick Chandel, High School
01/30/14 1133

IRB Approval: IRB approval indicates that this project meets the regulatory requirements for the protection of human subjects. IRB approval does not absolve the principal investigator from complying with other institutional, collegiate, or departmental policies or procedures.

Recruitment/Consent: Your IRB application has been approved for recruitment of subjects not to exceed the number indicated on your application form. If you are using written informed consent, the IRB-approved and stamped Informed Consent Document(s) are available in *my*IRB. The original signed Informed Consent Document should be placed in your research files. A copy of the Informed Consent Document should be given to the subject. (A copy of the *signed* Informed Consent Document should be given to the subject if your Consent contains a HIPAA authorization section.)

Continuing Review: Federal regulations require that the IRB re-approve research projects at intervals appropriate to the degree of risk, but no less than once per year. This process is called "continuing review." Continuing review for non-exempt research is required to occur as long as the research remains active for long-term follow-up of research subjects, even when the research is permanently closed to enrollment of new subjects and all subjects have completed all research-related interventions and to occur when the remaining research activities are limited to collection of private identifiable information. Your project "expires" at midnight on the date indicated on the preceding page ("Next IRB Approval Due on or Before"). You must obtain your next IRB approval of this project by that expiration date. You are responsible for submitting a Continuing Review application in sufficient time for approval before the expiration date, however you will receive reminder notice prior to the expiration date.

Modifications: Any change in this research project or materials must be submitted on a Modification application to the IRB for prior review and approval, except when a change is necessary to eliminate apparent immediate hazards to subjects. The investigator is required to promptly notify the IRB of any changes made without IRB approval to eliminate apparent immediate hazards to subjects using the Modification/Update Form. Modifications requiring the prior review and approval of the IRB include but are not limited to: changing the protocol or study procedures, changing investigators or funding sources, changing the Informed Consent Document, increasing the anticipated total number of subjects from what was originally approved, or adding any new materials (e.g., letters to subjects, ads, questionnaires).

Unanticipated Problems Involving Risks: You must promptly report to the IRB any unexpected adverse experience, as defined in the IRB/HRPO policies and procedures, and any other unanticipated problems involving risks to subjects or others. The Reportable Events Form (REF) should be used for reporting to the IRB.

Audits/Record-Keeping: Your research records may be audited at any time during or after the implementation of your project. Federal and University policies require that all research records be maintained for a period of seven (7) years following the close of the research project. For research that involves drugs or devices seeking FDA approval, the research records must be kept for a period of three years after the FDA has taken final action on the marketing application, if that is longer than seven years.

Additional Information: Complete information regarding research involving human subjects at Washington University is available in the "Washington University Institutional Review Board Policies and Procedures." Research investigators are expected to comply with these policies and procedures, and to be familiar with the University's Federalwide Assurance, the Belmont Report, 45CFR46, and other applicable regulations prior to conducting the research. This document and other important information is available on the HRPO website http://hrpohome.wustl.edu/.

Health Research Authority
NRES Committee East Midlands - Northampton
The Old Chapel
Royal Standard Place
Nottingham
NG1 6FS

Telephone: 0115 883 9440

23 January 2014

Dr Akash Deep
PICU, 3rd Floor Cheyne Wing
King's College Hospital, Denmark Hill
London
SE5 9RS

Dear Dr Deep,

**Study title:**	**Assessment of Worldwide Acute Kidney Injury (AKI), Renal Angina and Epidemiology**
**REC reference:**	**14/EM/0046**
**IRAS project ID:**	**146714**

Thank you for your letter of 22 January 2014, responding to the Proportionate Review Sub-Committee's request for changes to the documentation for the above study.

The revised documentation has been reviewed and approved by the sub-committee.

We plan to publish your research summary wording for the above study on the NRES website, together with your contact details, unless you expressly withhold permission to do so. Publication will be no earlier than three months from the date of this favourable opinion letter. Should you wish to provide a substitute contact point, require further information, or wish to withhold permission to publish, please contact the REC Manager Rebecca Morledge, NRESCommittee.EastMidlands-Northampton@nhs.net.

Confirmation of ethical opinion

On behalf of the Committee, I am pleased to confirm a favourable ethical opinion for the above research on the basis described in the application form, protocol and supporting documentation as revised.

Ethical review of research sites

The favourable opinion applies to all NHS sites taking part in the study, subject to management permission being obtained from the NHS/HSC R&D office prior to the start of the study (see "Conditions of the favourable opinion" below).

Conditions of the favourable opinion

The favourable opinion is subject to the following conditions being met prior to the start of the study.

Management permission or approval must be obtained from each host organisation prior to the start of the study at the site concerned.

Management permission ("R&D approval") should be sought from all NHS organisations involved in the study in accordance with NHS research governance arrangements.

Guidance on applying for NHS permission for research is available in the Integrated Research Application System or at http://www.rdforum.nhs.uk.

Where a NHS organisation's role in the study is limited to identifying and referring potential participants to research sites ("participant identification centre"), guidance should be sought from the R&D office on the information it requires to give permission for this activity.

For non-NHS sites, site management permission should be obtained in accordance with the procedures of the relevant host organisation.

Sponsors are not required to notify the Committee of approvals from host organisations.

Registration of Clinical Trials

All clinical trials (defined as the first four categories on the IRAS filter page) must be registered on a publically accessible database within 6 weeks of recruitment of the first participant (for medical device studies, within the timeline determined by the current registration and publication trees).

There is no requirement to separately notify the REC but you should do so at the earliest opportunity e.g when submitting an amendment. We will audit the registration details as part of the annual progress reporting process.

To ensure transparency in research, we strongly recommend that all research is registered but for non clinical trials this is not currently mandatory.

If a sponsor wishes to contest the need for registration they should contact Catherine Blewett (catherineblewett@nhs.net), the HRA does not, however, expect exceptions to be made. Guidance on where to register is provided within IRAS.

You should notify the REC in writing once all conditions have been met (except for site approvals from host organisations) and provide copies of any revised documentation with updated version numbers. The REC will acknowledge receipt and provide a final list of the approved documentation for the study, which can be made available to host organisations to facilitate their permission for the study. Failure to provide the final versions to the REC may cause delay in obtaining permissions.

It is the responsibility of the sponsor to ensure that all the conditions are complied with before the start of the study or its initiation at a particular site (as applicable).

Approved documents

The documents reviewed and approved by the Committee are:

Document	*Version*	*Date*
Investigator CV	Akash Deep	
Protocol	1 UK	08 January 2014
REC application	146714/548329/1/61	
Response to Request for Further Information		22 January 2014

Statement of compliance

The Committee is constituted in accordance with the Governance Arrangements for Research Ethics Committees and complies fully with the Standard Operating Procedures for Research Ethics Committees in the UK.

After ethical review

Reporting requirements

The attached document "After ethical review – guidance for researchers" gives detailed guidance on reporting requirements for studies with a favourable opinion, including:

- Notifying substantial amendments
- Adding new sites and investigators
- Notification of serious breaches of the protocol
- Progress and safety reports
- Notifying the end of the study

The NRES website also provides guidance on these topics, which is updated in the light of changes in reporting requirements or procedures.

Feedback

You are invited to give your view of the service that you have received from the National Research Ethics Service and the application procedure. If you wish to make your views known please use the feedback form available on the website.

Further information is available at National Research Ethics Service website > After Review

14/EM/0046	**Please quote this number on all correspondence**

We are pleased to welcome researchers and R & D staff at our NRES committee members' training days – see details at http://www.hra.nhs.uk/hra-training/

With the Committee's best wishes for the success of this project.

Yours sincerely,

pp

Mr Ken Willis
Chair

Email: NRESCommittee.EastMidlands-Northampton@nhs.net

Enclosures: *"After ethical review – guidance for researchers"*

Copy to: *The Research Office*

Dr Zoe Harris, King's College Hospital NHS Foundation Trust

Komite Etik Penelitian Kesehatan
Fakultas Kedokteran Universitas Indonesia
Rumah Sakit Cipto Mangunkusumo

Health Research Ethics Committee
Faculty of Medicine Universitas Indonesia
Cipto Mangunkusumo Hospital

Jalan Salemba Raya No. 6, Jakarta Pusat 10430. Telp. 021-3157008. E-mail: ec_fkui@yahoo.com

Nomor : 17 **/H2.F1/ETIK/2014**

KETERANGAN LOLOS KAJI ETIK

ETHICAL APPROVAL

Komite Etik Penelitian Kesehatan Fakultas Kedokteran Universitas Indonesia dalam upaya melindungi hak asasi dan kesejahteraan subyek penelitian kedokteran, telah mengkaji dengan teliti protokol berjudul:

The Ethics Committee of the Faculty of Medicine, University of Indonesia, with regards of the Protection of human rights and welfare in medical research, has carefully reviewed the *research protocol entitled:*

"Assessment of Worldwide Acute Kidney Injury (AKI) Renal Angina and Epidemiology (the AWARE Study)".

Peneliti Utama : **dr. Eka Laksmi, SpA(K)**
Principal Investigators

Nama Institusi : **Ilmu Kesehatan Anak FKUI/RSCM**
Name of the Institution

dan telah menyetujui protokol tersebut di atas.
and approved the above-mentioned protocol.

Jakarta 06 JAN 2014
Ketua
Chairman

Prof. Dr. dr. Rianto Setiabudy, SpFK

**Ethical approval* berlaku satu tahun dari tanggal persetujuan
**Peneliti berkewajiban

1. Menjaga kerahasiaan identitas subyek penelitian
2. Memberitahukan status penelitian apabila
   a. Setelah masa berlakunya keterangan lolos kaji etik, penelitian masih belum selesai, dalam hal ini *ethical clearance* harus diperpanjang
   b. Penelitian berhenti di tengah jalan
3. Melaporkan kejadian serius yang tidak diinginkan (*serious adverse events*)
4. Peneliti tidak boleh melakukan tindakan apapun pada subyek sebelum penelitian lolos kaji etik dan *informed consent*

January 24, 2014

BCM
Baylor College of Medicine

AYSE AKCAN ARIKAN
BAYLOR COLLEGE OF MEDICINE
PEDIATRICS: RENAL

Baylor College of Medicine
Office of Research
One Baylor Plaza, 600D
Houston, Texas 77030
Phone: (713) 798-6970
Fax: (713) 798-6990
Email: irb@bcm.tmc.edu

H-34082 - ASSESSMENT OF WORLDWIDE ACUTE KIDNEY INJURY (AKI), RENAL ANGINA AND EPIDEMIOLOGY(THE AWARE STUDY)

APPROVAL VALID FROM 1/24/2014 TO 12/3/2014

Dear Dr. AKCAN ARIKAN

The Institutional Review Board for Human Subject Research for Baylor College of Medicine and Affiliated Hospitals (BCM IRB) is pleased to inform you that the research protocol named above was approved.

The study may not continue after the approval period without additional IRB review and approval for continuation. You will receive an email renewal reminder notice prior to study expiration; however, it is your responsibility to assure that this study is not conducted beyond the expiration date.

Please be aware that only IRB-approved informed consent forms may be used when written informed consent is required.

Any changes in study or informed consent procedure must receive review and approval prior to implementation unless the change is necessary for the safety of subjects. In addition, you must inform the IRB of adverse events encountered during the study or of any new and significant information that may impact a research participants' safety or willingness to continue in your study.

The BCM IRB is organized, operates, and is registered with the United States Office for Human Research Protections according to the regulations codified in the United States Code of Federal Regulations at 45 CFR 46 and 21 CFR 56. The BCM IRB operates under the BCM Federal Wide Assurance No. 00000286, as well as those of hospitals and institutions affiliated with the College.

Sincerely yours,

Gabriel Habib

Association for the Accreditation of Human Research Protection Programs, Inc.
Full AAHRPP Accreditation

GABRIEL HABIB, M.D.
Institutional Review Board for Baylor College of Medicine and Affiliated Hospitals

ETHICAL COMMITTEE IN HEALTH RESEARCH
Dr. SOETOMO GENERAL HOSPITAL SURABAYA

"ETHICAL CLEARANCE"

305 / Panke.KKE / XII / 2013

THE ETHICAL COMMITTEE OF Dr. SOETOMO GENERAL HOSPITAL SURABAYA HAS BEEN REVIEWED THE SUBMITTED RESEARCH PROPOSAL THOROUGHLY, AND BY THIS LETTER, THE ETHICAL COMMITTEE IN HEALTH RESEARCH HEREBY DECLARES THE RESEARCH BY THE TITLE OF:

"Assessment of Worldwide Acute Kidney Injury (AKI), Renal Angina and Epidemiology (The Aware Study)"

PRINCIPAL INVESTIGATOR : Risky Vitria Prasetyo, dr., Sp.A (K)

SUPPORTING RESEARCHERS :
1. **Prof. M. Sjaifullah Noer, dr., SpA(K)**
2. **Dr. Ninik A. Soemyarso, MMPaed, dr., SpA(K)**
3. **Hari Kushartono, dr., SpA(K)**
4. **Abdul Latief Azis, dr., SpA(K)**
5. **Ira Dharmawati, dr., SpA**
6. **Dwi Putri Lestari, dr., SpA**
7. **Areta Idarto, dr**
8. **Heru Setiawan, dr**
9. **IDAA Shinta Kamaya, dr**
10. **Kristina Natalia Marbun, dr**

UNIT/ INSTITUTION/ RESEARCH LOCATION : Dr. Soetomo General Hospital Surabaya

IS DECLARED ETHICAL WORTHY.

SURABAYA, 10 DEC 2013
CHAIRMAN

Prof.Hari Sukanto, dr., Sp.KK (K)
NIP. 19471115 1973 03 1 001

Bambino Gesù
OSPEDALE PEDIATRICO

140 1869 2009
Vite che aiutano la vita

Il Comitato Etico

Gent.mo
Dott. Stefano Picca
Dialisi Pediatrica
SEDE

Roma, 21/01/2014
Prot. n. 19/RA

***Oggetto:** ASSESSMENT OF WORLDWIDE ACUTE KIDNEY INJURY (AKI), RENAL ANGINA AND EPIDEMIOLOGY*

***Protocollo:** AWARE STUDY*

Si trasmette l'estratto del verbale della riunione del 15/01/2014 di questo Comitato, relativo alla valutazione per lo studio in oggetto.

IL SEGRETARIO del CE
(Dr.ssa Chiara Mennini)

Piazza Sant'Onofrio, 4
00165 Roma
Tel. +39 06 6859 <inserire numero>
Fax +39 06 6859 <inserire numero>
e-mail <inserire indirizzo>@opbg.net

Bambino Gesù
Ospedale Pediatrico
Istituto di Ricovero e Cura
a Carattere Scientifico
www.ospedalebambinogesu.it

Joint Commission International

Bambino Gesù
OSPEDALE PEDIATRICO

140 1869 2009 Vite che aiutano la vita

Il Comitato Etico

ESTRATTO VERBALE

**Studio n°**	724 / 2013	**Arrivato il**	29-11-2013

Titolo ASSESSMENT OF WORLDWIDE ACUTE KIDNEY INJURY (AKI), RENAL ANGINA AND EPIDEMIOLOGY

Protocollo AWARE STUDY

Promotore Division of Nephrology and Hypertension Director, Center for Acute Care Nephrology Cincinnati Children's Hospital

Data Seduta **15/01/2013**

Il Comitato Etico, in osservanza a quanto previsto dal D.M. 15 luglio 1997; dalla Circolare n. 15 del 15 ottobre 2000 e dal D. Lgs. n. 211 del 24 giugno 2003, si è riunito per esaminare lo studio in oggetto ed in particolare la seguente documentazione:

Sperimentatori dello Studio

**Nome**	**Cognome**	**Unità Operativa**	**Sede**
**Stefano**	**Picca**	**Dialisi Pediatrica**	**P.zza S. Onofrio, 4 - 00165 Roma**

DOCUMENTAZIONE

	**Annotazioni**
Lettera di trasmissione	**Risposte post seduta**

Esito **PARERE FAVOREVOLE**

DELIBERAZIONE

Il Comitato Etico approva la richiesta di waiver del modulo di consenso per il trattamento dei dati personali.

Bambino Gesù
OSPEDALE PEDIATRICO

1869 2009
Vite che aiutano la vita

Il Comitato Etico

COMPONENTI DEL COMITATO

Prof. Rocco Agostino<br>*Neonatologo*	PRESENTE
Prof.ssa Maria Luisa Barbaccia<br>*Professore Ordinario di Farmacologia- Facoltà di Medicina e Chirurgia, Università "Tor Vergata"*	PRESENTE
Prof. Ignatio Carrasco De Paula<br>*Presidente della Pontificia Accademia Pro Vita*	PRESENTE
Prof. Cesare Colosimo<br>*Direttore Istituto di Radiologia, Università Cattolica del Sacro Cuore, Policlinico A. Gemelli*	NON CONVOCATO
Dr.ssa Tiziana Corsetti<br>*Responsabile della Farmacia, Ospedale Pediatrico Bambino Gesù*	ASSENTE
Prof Bruno Dallapiccola<br>*Direttore Scientifico dell'Ospedale Pediatrico Bambino Gesù*	PRESENTE
Prof.ssa Maria Luisa Di Pietro<br>*Professore Associato di Bioetica all'Università Cattolica del Sacro Cuore*	PRESENTE
Prof. Carlo Favaretti<br>*Presidente della Società Italiana di Health Technology Assessment*	NON CONVOCATO
Prof. P. Mastroiacovo<br>*Clinico Pediatra, Direttore International Center on Birth Defects*	PRESENTE
Prof. Giacinto Abele Donato Miggiano<br>*Direttore Centro Nutrizione Umana Facoltà di Medicina e Chirurgia dell'Università Cattolica*	NON CONVOCATO
Prof. Bruno Nobili<br>*Direttore del Dipartimento Assistenziale integrato Materno-infantile*<br>*Seconda Università degli Studi di Napoli*	PRESENTE
Dr. Valerio Nobili<br>*Resp. Struttura Complessa Malattie Epato-Metaboliche OPBG*	PRESENTE
Dr.ssa C. Offidani<br>*Medico Legale Ospedale Pediatrico Bambino Gesù*	PRESENTE
Prof.ssa Laura Palazzani<br>*Professore Ordinario Facoltà di Giurisprudenza, Università Lumsa*	ASSENTE
Dr. Claudio Pisanelli	PRESENTE

Il Comitato Etico

Farmacista A.O. San Filippo Neri

Dott.ssa Alessandra Querciati — ASSENTE
Coordinatore Infermieristico di Dipartimento-DPUO, , Ospedale Pediatrico Bambino Gesù

Dr.ssa L. Reali — PRESENTE
Pediatra di famiglia

Prof.ssa Carla Rossi — PRESENTE
Professore Ordinario di Statistica Medica, Policlinico Universitario "Tor Vergata"

Dr.ssa Stella Scalamandrè — PRESENTE
Esperto Giuridico-Legale OPBG

Dott.ssa Gianna Scarpelli — PRESENTE
Membro Associazione Volontari, Ospedale Pediatrico Bambino Gesù

PRESIDENTE
Prof. P. Mastroiacovo
Clinico Pediatra, Direttore International Center on Birth Defects

SEGRETARIO
Dr.ssa C. Mennini
Resp. Segreteria Tecnico Scientifica, Ospedale Pediatrico Bambino Gesù – Roma

IL SEGRETARIO del CE
(Dr.ssa Chiara Mennini)

Bambino Gesù
OSPEDALE PEDIATRICO

1869 2009
Vite che aiutano la vita

Il Comitato Etico

Gent.mo
Dott. Stefano Picca
Dialisi Pediatrica

SEDE

Roma, 27/12/2013
Prot. n. 1082 LB

Oggetto: ASSESSMENT OF WORLDWIDE ACUTE KIDNEY INJURY (AKI), RENAL ANGINA AND EPIDEMIOLOGY

Protocollo: AWARE STUDY

Si trasmette l'estratto del verbale della riunione del 11/12/2013 di questo Comitato, relativo alla valutazione dello studio in oggetto.

IL SEGRETARIO del CE
(Dr.ssa Chiara Mennini)

Piazza Sant'Onofrio, 4
00165 Roma
Tel. +39 06 6859 <inserire numero>
Fax +39 06 6859 <inserire numero>
e-mail <inserire indirizzo>@opbg.net

Bambino Gesù
Ospedale Pediatrico
Istituto di Ricovero e Cura
a Carattere Scientifico
www.ospedalebambinogesu.it

Joint Commission International

Bambino Gesù
OSPEDALE PEDIATRICO

Il Comitato Etico

ESTRATTO VERBALE

Studio n° 724 / 2013 **Arrivato il** 29-11-2013

Titolo ASSESSMENT OF WORLDWIDE ACUTE KIDNEY INJURY (AKI), RENAL ANGINA AND EPIDEMIOLOGY

Protocollo AWARE STUDY **Eudract**

Farmaco

Area Terapeutica	Nome commerciale	Principio Attivo
**Area Terapeutica**	**Farmaco**	**Principio Attivo**

Promotore Division of Nephrology and Hypertension Director, Center for Acute Care Nephrology Cincinnati Childr

Data Seduta **11 dicembre 2013**

Il Comitato Etico, in osservanza a quanto previsto dal D.M. 15 luglio 1997; dalla Circolare n. 15 del 15 ottobre 2000 e dal D. Lgs. n. 211 del 24 giugno 2003, si è riunito per esaminare lo studio in oggetto ed in particolare la seguente documentazione:

Sperimentatori dello Studio

Nome	Cognome	Unità Operativa	Sede
**Stefano**	**Picca**	**Dialisi Pediatrica**	**P.zza S.Onofrio,4 - 00165 Roma**

DOCUMENTAZIONE

		Ver	Data ver.
Richiesta di parere			**10-11-2013**
Scheda Finanziaria			
Sinossi		**1.0**	**21-11-2013**
Lettera di trasmissione			**21-11-2013**
Protocollo		**1.0**	
Documentazione aggiuntiva	Memorandum 01		**28-10-2013**
Documentazione aggiuntiva	Manual of Operating Procedures	**1.0**	**04-11-2013**
Documentazione aggiuntiva	Approval Letter CCHMC		**28-10-2013**

Esito **PARERE FAVOREVOLE**

Piazza Sant'Onofrio, 4
00165 Roma
Tel. +39 06 68592572
Fax +39 06 68594547
e-mail chiara.mennini@opbg.net

Bambino Gesù
Ospedale Pediatrico
Istituto di Ricovero e Cura
a Carattere Scientifico
www.ospedalebambinogesu.it

Joint Commission International

Il Comitato Etico

DELIBERAZIONE

Il Comitato Etico approva lo studio con la richiesta di prevedere un modulo di consenso per il trattamento dei dati personali.

Dei documenti modificati/richiesti come sopra non sarà necessario che il CE li riapprovi ma sarà sufficiente far pervenire copia alla Segreteria del CE

COMPONENTI DEL COMITATO

Prof. Rocco Agostino<br>*Neonatologo*	PRESENTE
Prof.ssa Maria Luisa Barbaccia<br>*Professore Ordinario di Farmacologia- Facoltà di Medicina e Chirurgia, Università "Tor Vergata"*	PRESENTE
Prof. Ignatio Carrasco De Paula<br>*Presidente della Pontificia Accademia Pro Vita*	PRESENTE
Prof. Cesare Colosimo<br>*Direttore Istituto di Radiologia,*<br>*Università Cattolica del Sacro Cuore, Policlinico A. Gemelli*	NON CONVOCATO
Dr.ssa Tiziana Corsetti<br>*Responsabile della Farmacia, Ospedale Pediatrico Bambino Gesù*	ASSENTE
Prof Bruno Dallapiccola<br>*Direttore Scientifico dell'Ospedale Pediatrico Bambino Gesù*	PRESENTE
Prof.ssa Maria Luisa Di Pietro<br>*Professore Associato di Bioetica all'Università Cattolica del Sacro Cuore*	PRESENTE
Prof. Carlo Favaretti<br>*Presidente della Società Italiana di Health Technology Assessment*	NON CONVOCATO
Prof. P. Mastroiacovo<br>*Professore di Pediatria, Direttore International Center on Birth Defects*	PRESENTE
Prof. Giacinto Abele Donato Miggiano<br>*Direttore Centro Nutrizione Umana*<br>*Facoltà di Medicina e Chirurgia dell'Università Cattolica*	NON CONVOCATO
Prof. Bruno Nobili<br>*Direttore del Dipartimento Assistenziale integrato Materno-infantile*	PRESENTE

Bambino Gesù
OSPEDALE PEDIATRICO

140 1869 2009
Vite che aiutano la vita

Il Comitato Etico

Seconda Università degli Studi di Napoli

Dr. Valerio Nobili<br>*Resp. Struttura Complessa Malattie Epato-Metaboliche OPBG*	PRESENTE
Dr.ssa C. Offidani<br>*Medico Legale Ospedale Pediatrico Bambino Gesù*	PRESENTE
Prof.ssa Laura Palazzani<br>*Professore Ordinario Facoltà di Giurisprudenza, Università Lumsa*	PRESENTE
Dr. Claudio Pisanelli<br>*Farmacista A.O. San Filippo Neri*	PRESENTE
Dott.ssa Alessandra Querciati<br>*Coordinatore Infermieristico di Dipartimento-DPUO, , Ospedale Pediatrico Bambino Gesù*	ASSENTE
Dr.ssa L. Reali<br>*Pediatra di famiglia*	PRESENTE
Dr.ssa C. Offidani per il Dr. M. Raponi<br>*Direttore Sanitario dell'Ospedale Pediatrico Bambino Gesù*	PRESENTE
Prof.ssa Carla Rossi<br>*Professore Ordinario di Statistica Medica, Policlinico Universitario "Tor Vergata"*	PRESENTE
Dr.ssa Stella Scalamandrè<br>*Esperto Giuridico- Legale OPBG*	PRESENTE
Dott.ssa Gianna Scarpelli<br>*Membro Associazione Volontari, Ospedale Pediatrico Bambino Gesù*	PRESENTE

PRESIDENTE
Prof. P. Mastroiacovo
Clinico Pediatra, Direttore International Center on Birth Defects

SEGRETARIO
Dr.ssa C. Mennini
Resp. Segreteria Tecnico Scientifica, Ospedale Pediatrico Bambino Gesù – Roma

IL SEGRETARIO del CE
(Dr.ssa Chiara Mennini)

Piazza Sant'Onofrio, 4
00165 Roma
Tel +39 06 68592572
Fax +39 06 68594547
e-mail chiara.mennini@opbg.net

EMORY UNIVERSITY | Institutional Review Board

TO: Matthew Paden, MD
Principal Investigator
Crit Care

DATE: January 14, 2014

RE: **Expedited Approval**
IRB00071031
Assessment of Worldwide Acute Kidney Injury (AKI), Renal Angina and Epidemiology

Thank you for submitting a new application for this protocol. This research is eligible for expedited review under 45 CFR.46.110 and/or 21 CFR 56.110 because it poses minimal risk and fits the regulatory categories F[3, 5] as set forth in the Federal Register. The Emory IRB reviewed it by expedited process on 01/14/2014 and granted approval effective from **01/14/2014** through **01/13/2015.** Thereafter, continuation of human subjects research activities requires the submission of a renewal application, which must be reviewed and approved by the IRB prior to the expiration date noted above. Please note carefully the following items with respect to this approval:

- A complete HIPAA/consent waiver was granted.

The following documents were included in this review:

- AWARE_Master Protocol V1, version date 10/28/2013

Any reportable events (e.g., unanticipated problems involving risk to subjects or others, noncompliance, breaches of confidentiality, HIPAA violations, protocol deviations) must be reported to the IRB according to our Policies & Procedures at www.irb.emory.edu, immediately, promptly, or periodically. Be sure to check the reporting guidance and contact us if you have questions. Terms and conditions of sponsors, if any, also apply to reporting.

Before implementing any change to this protocol (including but not limited to sample size, informed consent, and study design), you must submit an amendment request and secure IRB approval.

In future correspondence about this matter, please refer to the IRB file ID, name of the Principal Investigator, and study title. Thank you

Olga Dashevskaya, JD

SPECTRUM HEALTH
Human Research Protection Program
Office of the Institutional Review Board
100 Michigan NE, MC 038
Grand Rapids, MI 49503
616.486.2031
irb@spectrumhealth.org
www.spectrumhealth.org/HRPP

APPROVAL OF RESEARCH

January 14, 2014

Richard Hackbarth MD
Helen DeVos Children's Hospital
100 Michigan St. NE
MC 117
Grand Rapids, MI 49503

TYPE OF REVIEW: **Initial, Non-Committee Review**

IRB#: **2013-284** *(please reference this number in all correspondence with the IRB)*

PROTOCOL NAME: **Assessment of Worldwide Acute Kidney Injury (AKI), Renal Angina and Epidemiology**

SPONSOR: **Cincinnati Children's Hospital Medical Center**

Dear Dr. Hackbarth:

The above referenced protocol and associated materials were reviewed and approved by the IRB via expedited review on January 13, 2014 under categories 3 & 5 as described in 45 CFR 46.110.

The approval period for this research is from **January 13, 2014** to **January 12, 2015.**

The IRB reviewed the following documents related to the approval of the research proposal:

- Initial application signed 11/07/13
- Study protocol dated 10/28/13
- Case Report Form V2.0 dated 01/10/14
- Manual of Operating Procedures V3.0 dated 01/10/14
- Memorandum from Primary Site dated 10/28/13
- Memorandum from Primary Site dated 01/10/14
- CCHMC Approval letter dated 10/28/13

The IRB made the following determinations:

1. **WAIVER OF CONSENT/HIPAA AUTHORIZATION**: A waiver of consent has been approved per 45 CFR 46.116(d) and a waiver of HIPAA authorization has been approved per 45 CFR 164.512(i)(2)(ii).

2. **RESEARCH INVOLVING CHILDREN**: The inclusion of children has been approved per 45 CFR 46.404 / 21 CFR 50.51.

3. **WAIVER OF PARENTAL PERMISSION**: A waiver of parental permission has been approved per 45 CFR 46.116(d).

4. **WAIVER OF ASSENT**: A waiver of assent has been approved per 45 CFR 46.116(d).

Any changes made to the study following this approval, including informed consent changes, require submission in writing to the IRB and approval by the committee. Changes may not be implemented until approved by the IRB except when necessary to eliminate apparent immediate hazards to the subject. Approval of your research means you are responsible for complying with all applicable policies and procedures of the FDA, OHRP, HIPAA, Spectrum Health, and the Spectrum Health IRB. Also, please be advised that unanticipated problems involving risk to subjects or others must be *promptly* reported to the IRB. You may reference the Investigator Manual for guidance on expectations of the IRB after approval.

The IRB requires submission of the Continuing Review Progress Report or Study Completion Notification to the committee prior to the study expiration date. It is recommended you submit this xform 4-6 weeks prior to the expiration date to allow time for processing. Your study approval expires on **January 12, 2015 at 11:59pm** and cannot continue until re-approved by the Spectrum Health IRB. If your study has been completed, terminated, or if you do not wish to continue, please submit the Study Completion Notification before the expiration date.

If you have any questions please contact the Spectrum Health IRB office at 616-486-2031, email irbassist@spectrumhealth.org, or visit us on the web at www.spectrumhealth.org/HRPP.

Sincerely,

J. Jones

Jeffrey Jones MD
Chair, Spectrum Health IRB

cc: Akunne Ndika MBBS, MPH

The Sydney children's Hospitals Network

care, advocacy, research, education

Corner Hawkesbury Road
and Hainsworth Street
Locked Bag 4001
Westmead NSW 2145
Sydney Australia
DX 8213 Parramatta
Tel +61 2 9845 0000
Fax +61 2 9845 3489
http://www.schn.health.nsw.gov.au/
ABN 53 188 579 090

Contact for this correspondence:
Research and Development
Name: Sabine Giesebrecht
Phone: (02) 9845 3084
Facsimile: (02) 9845 1317
Email: sabine.giesebrecht@health.nsw.gov.au

Date: 08 January 2014

Dr Deirdre Hahn
Nephrology
CHW-Westmead

Site Authorisation Letter

Dear Dr Hahn,

**HREC reference number:**	**LNR/13/SCHN/455**
**SSA reference number:**	**LNRSSA/13/SCHN/456**
**Project title:**	**Assessment of World Wide Acute Kidney Injury, Renal Angina and Epidemiology (The AWARE study)**
**Site:**	**The Children's Hospital at Westmead**

Thank you for submitting an application for authorisation of this project. I am pleased to inform you that authorisation has been granted for this study to take place at the above site.

The following conditions apply to this research project. These are additional to those conditions imposed by the Human Research Ethics Committee that granted ethical approval:

1. Please advise us of the date when the project starts at this site.
2. Proposed amendments to the research protocol or conduct of the research which may affect the ethical acceptability of the project, and which are submitted to the lead HREC for review, are copied to the research governance officer.
3. Proposed amendments to the research protocol or conduct of the research which may affect the ongoing site acceptability of the project are to be submitted to the research governance officer.

Yours sincerely,

Sabine Giesebrecht
Research Governance Officer

The Sydney children's Hospitals Network

care, advocacy, research, education

Contact for this correspondence:
Research and Development
Ethics & Governance Administration Assistant
Phone: (02) 9845 1253
Facsimile: (02) 9845 1317
Email: ethics.schn@health.nsw.gov.au

Corner Hawkesbury Road
and Hainsworth Street
Locked Bag 4001
Westmead NSW 2145
Sydney Australia
DX 8213 Parramatta
Tel +61 2 9845 0000
Fax +61 2 9845 3489
http://www.schn.health.nsw.gov.au/
ABN 53 188 579 090

6 January 2014

Dr Sean Kennedy
Nephrology
Children's Hospital at Westmead

Dear Dr Kennedy,

HREC Reference: **LNR/13/SCHN/455**

Project title: **Assessment of World Wide Acute Kidney Injury, Renal Angina and Epidemiology (The AWARE study).**

Reviewed for: **Sydney Children's Hospital Network**

Thank you for submitting the above project for single ethical and scientific review. This project was first considered out of session by the Executive of the Sydney Children's Hospitals Network Human Research Ethics Committee (HREC). This HREC has been accredited by the NSW Department of Health as a lead HREC under the model for single ethical and scientific review.

This lead HREC is constituted and operates in accordance with the National Health and Medical Research Council's *National Statement on Ethical Conduct in Human Research* and *CPMP/ICH Note for Guidance on Good Clinical Practice.*

I am pleased to advise that the HREC Executive granted ethical approval of this research project. **Your approval is valid from the date of this letter.**

The documents reviewed and approved include:

LNR application form – Submission code AU/6/1355116		6/11/2013
The AWARE Study Master Protocol	V 1	28/10/2013
Cover letter		18/11/2013
Parent/Guardian Information Sheet and Consent Form	V 1	18/11/2013

Please note the following conditions of approval:

1. The co-ordinating investigator will immediately report anything which might warrant review of ethical approval of the project in the specified format, including:
   - Unforeseen events that might affect continued ethical acceptability of the project.

2. Proposed changes to the research protocol, conduct of the research, or length of HREC approval, will be provided to the HREC for review in the specified format.

3. The HREC will be notified, giving reasons, if the project is discontinued at a site before the expected date of completion.

4. The co-ordinating investigator will provide an annual report to the HREC and at completion of the study. The annual report form is available on the Hospital's intranet and internet or from the Secretary.

5. Your approval is valid for 5 years from the date of the final approval letter. If your project extends beyond five years then at the 5 year anniversary you are required to resubmit your protocol, according to the latest guidelines, seeking the renewal of your previous approval. In the event of a project **not having commenced** within 12 months of its approval, the approval will lapse and reapplication to the HREC will be required.

Should you have any queries about the HREC's consideration of your project please contact the Ethics and Governance Administration Assistant on 9845 1253.

You are reminded that this letter constitutes ethical approval only. You must not commence this research project at a site until separate authorisation from the Chief Executive or delegate of that site has been obtained.

A copy of this letter must be forwarded to all site investigators for submission to the relevant Research Governance Officer.

The HREC wishes you every success in your research.

Yours faithfully

Ms Jillian Shute
Executive Officer
Sydney Children's Hospitals Network Human Research Ethics Committee

c.c.: Dr Diedre Hahn, CHW

南京医科大学附属南京儿童医院医学伦理委员会

Nanjing Children's Hospital

科研课题伦理审批件

编号：201312030

科研课题名称	全球多中心ICU儿童急性肾损伤流行病学调查及标记分析筛选项目实施方		
课题任务来源	自主研究	课题编号	无
项目负责人	黄松明、张爱华	项目负责人所在单位	南京医科大学附属南京儿童医院肾脏科
会议地点	南京医科大学附属南京儿童医院行政楼5楼会议室	审批日期	2013年12月24日
主任委员	金福年	副主任委员	莫绪明

审批内容：

1、伦理审查申请表

2、研究方案

3、受试者知情同意书

出席人数：应到15人，实到10人

表决情况：同意9人，修改后同意 人，不同意 人，回避1人

伦理委员会综合意见：

1、研究者资格及专业条件： √符合 □不符合

2、研究方案： √适当 □需修改 □不适当

3、受试者入选方法： √适当 □需修改 □不适当

4、知情同意书告知部分： √适当 □需修改 □不适当

5、知情同意书签字部分： √适当 □需修改 □不适当

审批结论：

1、√同意 2、作必要修改后同意 3、不同意 4、终止或暂停先前批准的试验

其他：

*依据GCP要求及相关法规，本伦理委员会的组织和实施相对独立

*本院伦理委员会的人员组成和工作程序是符合GCP原则以及国家相关规定的

南京医科大学附属南京儿童医院医学伦理委员会（盖章）

（印章：南京医科大学附属南京儿童医院伦理委员会）

二〇一三年十二月二十四日

UBC C&W Research Ethics Board
A2-136, 950 West 28th Avenue
Vancouver, BC V5Z 4H4
Tel: (604) 875-3103 Fax: (604) 875-2496
Email: cwreb@cw.bc.ca
Website: http://www.cfri.ca/reb

ETHICS CERTIFICATE OF DELEGATED APPROVAL

PRINCIPAL INVESTIGATOR:	INSTITUTION / DEPARTMENT:	UBC C&W NUMBER:
Cherry Mammen	UBC/Medicine, Faculty of/Paediatrics	H13-03154

INSTITUTION(S) WHERE RESEARCH WILL BE CARRIED OUT:

Institution	Site
Children's and Women's Health Centre of BC (incl. Sunny Hill)	Child & Family Research Institute

Other locations where the research will be conducted:
N/A

CO-INVESTIGATOR(S):
Peter Skippen

SPONSORING AGENCIES:
N/A

PROJECT TITLE:
Assessment of Worldwide AKI Renal Angina and Epidemiology

REMINDER: The current UBC Children's and Women's approval for this study expires: December 23, 2014

The UBC Children's and Women's Research Ethics Board Minimal Risk Committee has reviewed the above described research project, including associated documentation noted below, and finds the research project acceptable on ethical grounds for research involving human subjects and hereby grants ethics approval. Note that institutional approval is required before research can commence at C&W.

This approval applies to research ethics issues only. The approval does not obligate an institution or any of its departments to proceed with activation of the study. The Principal Investigator for the study is responsible for identifying and ensuring that resource impacts from this study on any institution are properly negotiated, and that other institutional policies are followed. The REB assumes that investigators and the coordinating office of all trials continuously review new information for findings that indicate a change should be made to the protocol, consent documents or conduct of the trial and that such changes will be brought to the attention of the REB in a timely manner.

DOCUMENTS INCLUDED IN THIS APPROVAL: | **APPROVAL DATE:** December 23, 2013

Document Name	Version	Date
**Protocol:**		
AWARE protocol	1	October 28, 2013

**Other Documents:**		
AWARE Case Report Form	1	November 18, 2013

CERTIFICATION:
In respect of clinical trials:
1. The membership of this Research Ethics Board complies with the membership requirements for Research Ethics Boards defined in Division 5 of the Food and Drug Regulations.
2. The Research Ethics Board carries out its functions in a manner consistent with Good Clinical Practices.
3. This Research Ethics Board has reviewed and approved the clinical trial protocol and informed consent form for the trial which is to be conducted by the qualified investigator named above at the specified clinical trial site. This approval and the views of this Research Ethics Board have been documented in writing.

The documentation included for the above-named project has been reviewed by the UBC Children's and Women's Research Ethics Board, and the research study, as presented in the documentation, was found to be acceptable on ethical grounds for research involving human subjects and was approved by the UBC Children's and Women's Research Ethics Board.

Approved by one of:

Dr. Marc Levine, Chair **Dr. Caron Strahlendorf, Associate Chair**

STANFORD UNIVERSITY

Stanford, CA 94305 [Mail Code 5579]

David D Oakes, M.D. (650) 723-5215

CHAIR, PANEL ON MEDICAL HUMAN SUBJECTS (650) 725-8013

Certification of Human Subjects Approvals

Date: December 17, 2013

To: Scott Sutherland, Pediatrics - Nephrology
Kimberly J Sands

From: David D Oakes, M.D., Administrative Panel on Human Subjects in Medical Research

Protocol Assessment of Worldwide Acute Kidney Injury (AKI), Renal Angina, and Epidemiology (The AWARE Study)

Protocol ID: 29516 **IRB Number:** 6208 (Panel: 8)

The IRB approved human subjects involvement in your research project on 12/17/2013. **'Prior to subject recruitment and enrollment, if this is: a Cancer-related study, you must obtain Cancer Center Scientific Review Committee (SRC) approval;** a CTRU study, you must obtain CTRU approval; a VA study, you must obtain VA R and D Committee approval; and if a contract is involved, it must be signed.'

The expiration date of this approval is 11/30/2016 at Midnight. If this project is to continue beyond that date, you must submit an updated protocol in advance for the IRB's re-approval. If this protocol is used in conjunction with any other human use it must be re-approved. Proposed changes to approved research must be reviewed and approved prospectively by the IRB. No changes may be initiated without prior approval by the IRB, except where necessary to eliminate apparent immediate hazards to subjects. (Any such exceptions must be reported to the IRB within 10 working days.) Unanticipated problems involving risks to participants or others and other events or information, as defined and listed in the Report Form, must be submitted promptly to the IRB. (See Events and Information that Require Prompt Reporting to the IRB at http://humansubjects.stanford.edu.)

All continuing projects and activities must be reviewed and re-approved on or before Midnight of the expiration date. The approval period will be less than one year if so determined by the IRB. It is your responsibility to resubmit the project to the IRB for continuing review and to report the completion of the protocol to the IRB within 30 days.

Please remember that all data, including all signed consent form documents, must be retained for a minimum of three years past the completion of this research. Additional requirements may be imposed by your funding agency, your department, or other entities. (See Policy on Retention of and Access to Research Data at http://stanford.edu/dept/DoR/rph/2-10.html.)

This institution is in compliance with requirements for protection of human subjects, including 45 CFR 46, 21 CFR 50 and 56, and 38 CFR 16.

David D Oakes, M.D., Chair

Approval Period: 12/17/2013 THROUGH 11/30/2016

Review Type: CHART REVIEW - NEW

Funding: None

1

Assurance Number: FWA00000935 (SU), FWA00000933 (LPCH)

THE UNIVERSITY OF ALABAMA AT BIRMINGHAM

Institutional Review Board for Human Use

Form 4: IRB Approval Form
Identification and Certification of Research
Projects Involving Human Subjects

UAB's Institutional Review Boards for Human Use (IRBs) have an approved Federalwide Assurance with the Office for Human Research Protections (OHRP). The Assurance number is FWA00005960 and it expires on January 24, 2017. The UAB IRBs are also in compliance with 21 CFR Parts 50 and 56.

Principal Investigator: ASKENAZI, DAVID

Co-Investigator(s):

Protocol Number: **X131203018**

Protocol Title: *Assessment of Worldwide Acute Kidney Injury (AKI), Renal Angina and Epidemiology (The AWARE Study)*

The IRB reviewed and approved the above named project on 12-17-13. The review was conducted in accordance with UAB's Assurance of Compliance approved by the Department of Health and Human Services. This Project will be subject to Annual continuing review as provided in that Assurance.

This project received EXPEDITED review.

IRB Approval Date: 12-17-13

Date IRB Approval Issued: 12-17-13

IRB Approval No Longer Valid On: 12-17-14

HIPAA Waiver Approved?: Yes

Partial HIPAA Waiver Approved?: N/A

Marilyn Doss

Marilyn Doss, M.A.
Vice Chair of the Institutional Review Board for Human Use (IRB)

Investigators please note:

The IRB approved consent form used in the study must contain the IRB approval date and expiration date.

IRB approval is given for one year unless otherwise noted. For projects subject to annual review research activities may not continue past the one year anniversary of the IRB approval date.

Any modifications in the study methodology, protocol and/or consent form must be submitted for review and approval to the IRB prior to implementation.

Adverse Events and/or unanticipated risks to subjects or others at UAB or other participating institutions must be reported promptly to the IRB.

470 Administration Building
701 20th Street South
205.934.3789
Fax 205.934.1301
irb@uab.edu

The University of
Alabama at Birmingham
Mailing Address:
AB 470
1720 2ND AVE S
BIRMINGHAM AL 35294-0104

THE UNIVERSITY OF ALABAMA AT BIRMINGHAM

Institutional Review Board for Human Use

PI: ASKENAZI, DAVID

Protocol # X131203018

UAB IRB Approval of Waiver of Informed Consent and/or Waiver of Patient Authorization

☒ **Approval of Waiver of Informed Consent to Participate in Research.** The IRB reviewed the proposed research and granted the request for waiver of informed consent to participate in research, based on the following findings:

1. The research involves no more than minimal risk to the subjects.
2. The research cannot practicably be carried out without the waiver.
3. The waiver will not adversely affect the rights and welfare of the subjects.
4. When appropriate, the subjects will be provided with additional pertinent information after participation.

Check one:
- ☒ **and** Waiver of Authorization (below)
- ☐ **or** Waiver of Authorization (below)
- ☐ Waiver of Authorization not applicable

☒ **Approval of Waiver of Patient Authorization to Use PHI in Research.** The IRB reviewed the proposed research and granted the request for waiver of patient authorization to use PHI in research, based on the following findings:

1. The use/disclosure of PHI involves no more than minimal risk to the privacy of individuals
   - i. There is an adequate plan to protect the identifiers from improper use and disclosure.
   - ii. There is an adequate plan to destroy the identifiers at the earliest opportunity consistent with conduct of the research, unless there is a health or research justification for retaining the identifiers or such retention that is otherwise required by law.
   - iii. There is an assurance that the PHI will not be reused or disclosed to any other person or entity, except as required by law, for authorized oversight of the research study, or for other research for which the use or disclosure of PHI would be permitted.
2. The research cannot practicably be conducted without the waiver or alteration.
3. The research cannot practicably be conducted without access to and use of the PHI.

—OR—

☐ **Full Review**

The IRB reviewed the proposed research at a **convened meeting** at which a majority of the IRB was present, including one member who is not affiliated with any entity conducting or sponsoring the research, and not related to any person who is affiliated with any of such entities. The waiver of authorization was approved by the majority of the IRB members present at the meeting.

Date of Meeting

Signature of Chair, Vice-Chair or Designee

Date

☒ **Expedited Review**

The IRB used an **expedited review procedure** because the research involves no more than minimal risk to the privacy of the individuals who are the subject of the PHI for which use or disclosure is being sought. The review and approval of the waiver of authorization were carried out by the Chair of the IRB, or by one of the Vice-Chairs of the IRB as designated by the Chair of the IRB.

12-17-13

Date of Expedited Review

Marilyn Doss

Signature of Chair, Vice-Chair or Designee

Date 12-17-13

470 Administration Building
701 20th Street South
205.934.3789
Fax 205.934.1301
irb@uab.edu

The University of Alabama at Birmingham
Mailing Address:
AB 470
1720 2ND AVE S
BIRMINGHAM AL 35294-0104

Alan Sugar

Cc:

Susan Hieber
David Selewski
Kera Luckritz

Subject: Initial Study Approval for [HUM00082719]

SUBMISSION INFORMATION:
Study Title: The AWARE Study
Full Study Title (if applicable): Assessment of Worldwide Actue Kidney INjury (AKI), Renal Angina and Epidemiology
Study eResearch ID: HUM00082719
Date of this Notification from IRB: 12/17/2013
Review: Expedited
Initial IRB Approval Date: 12/16/2013
Current IRB Approval Period: 12/16/2013 - 12/15/2014
Expiration Date: Approval for this expires at **11:59 p.m. on 12/15/2014**
UM Federalwide Assurance (FWA): FWA00004969 (For the current FWA expiration date, please visit the UM HRPP Webpage)
OHRP IRB Registration Number(s): IRB00005467

Approved Risk Level(s):

Name	Risk Level
HUM00082719	No more than minimal risk

NOTICE OF IRB APPROVAL AND CONDITIONS:
The IRBMED has reviewed and approved the study referenced above. The IRB determined that the proposed research conforms with applicable guidelines, State and federal regulations, and the University of Michigan's Federalwide Assurance (FWA) with the Department of Health and Human Services (HHS). You must conduct this study in accordance with the description and information provided in the approved application and associated documents.

APPROVAL PERIOD AND EXPIRATION:
The approval period for this study is listed above. Please note the expiration date. If the approval lapses, you may not conduct work on this study until appropriate approval has been re-established, except as necessary to eliminate apparent immediate hazards to research subjects. Should the latter occur, you must notify the IRB Office as soon as possible.

IMPORTANT REMINDERS AND ADDITIONAL INFORMATION FOR INVESTIGATORS

APPROVED STUDY DOCUMENTS:
You must use any date-stamped versions of recruitment materials and informed consent documents available in the eResearch workspace (referenced above). Date-stamped materials are available in the "Currently Approved Documents" section on the "Documents" tab.

RENEWAL/TERMINATION:
At least two months prior to the expiration date, you should submit a continuing review application either to renew or terminate the study. Failure to allow sufficient time for IRB review may result in a lapse of approval that may also affect any funding associated with the study.

AMENDMENTS:
All proposed changes to the study (e.g., personnel, procedures, or documents), must be approved in advance by the IRB through the amendment process, except as necessary to eliminate apparent immediate hazards to research subjects. Should the latter occur, you must notify the IRB Office as soon as possible.

AEs/ORIOs:
You must inform the IRB of all unanticipated events, adverse events (AEs), and other reportable information and occurrences (ORIOs). These include but are not limited to events and/or information that may have physical, psychological, social, legal, or economic impact on the research subjects or other.

Investigators and research staff are responsible for reporting information concerning the approved research to the IRB in a timely fashion, understanding and adhering to the reporting guidance (http://www.med.umich.edu/irbmed/ae_orio/index.htm), and not implementing any changes to the research without IRB approval of the change via an amendment submission. When changes are necessary to eliminate apparent immediate hazards to the subject, implement the change and report via an ORIO and/or amendment submission within 7 days after the action is taken. This includes all information with the potential to impact the risk or benefit assessments of the research.

SUBMITTING VIA eRESEARCH:
You can access the online forms for continuing review, amendments, and AEs/ORIOs in the eResearch workspace for this approved study (referenced above).

MORE INFORMATION:
You can find additional information about UM's Human Research Protection Program (HRPP) in the Operations Manual and other documents available at: www.research.umich.edu/hrpp.

**Michael Geisser**	**Alan Sugar**
**Co-chair, IRBMED**	**Co-chair, IRBMED**

****

Electronic Mail is not secure, may not be read every day, and should not be used for urgent or sensitive issues

SNUMC/SNUHIRB
Seoul National University College of Medicine/Seoul National University Hospital
Institutional Review Board
Tel :82-2-2072-0694
FAX:82-2-2072-0368 101 Daehak-ro, Jongno-gu, Seoul, 110-744, Korea

Certificate of Approval

THE FOLLOWING WERE APPORVED:

BOARD ACTION DATED: 20 Nov 2013

STUDY NO :

IRB NO: H-1311-011-527

INVESTIGATOR: IL SOO HA

SPONSOR:

PROTOCOL NO:

TITLE: Assessment of Worldwide Acute Kidney Injury (AKI), Renal Angina and Epidemiology

APPROVAL INCLUDES:

1. Protocol
2. Waiver of Informed Consent Form
3. Informed Consent Form for Human-Derived Material Research
4. Case Report Form
5. Investigator's Brochuer
6. Principal investigator's CV

ALL CONDITIONS OF APPROVAL PREVIOUSLY ESTABLISHED BY SNUMC/SNUHIRB FOR THIS RESEARCH PROJECT CONTINUE TO APPLY.

CONTINUING REVIEW REPORT INTERVAL: Annually

IF YOU HAVE ANY QUESTIONS, CONTACT SNUMC/SNUH IRB (Tel: 82-2-2072-0694)

This is to certify that the information contained herein is true and correct as reflected in the records of the SNUMC/SNUH Institutional Review Board. **We certify that SNUMC/SNUH IRB is in full compliance with Good Clinical Practice as defined under the Korea Food and Drug Administration (KFDA) regulations and the International Conference on Harmonisation (ICH) guidelines.**

Jaesung Park

20 Nov 2013

Chairperson **Date**

ALL SNUMC/SNUH IRB APPROVED INVESTIGATORS MUST COMPLY WITH THE FOLLOWING:

1. Conduct the research as required by the protocol.
2. Use only the Consent Form bearing the SNUMC/SNUH IRB " APPROVED" stamp.
3. Provide non-Korean speaking subjects with a certified translation of the approved Consent Form in the subject's first language. The translated version must be approved by the SNUMC/SNUH IRB.
4. Obtain pre-approval from the SNUMC/SNUH IRB of any changes in the research activity (except when necessary to protect human subjects; immediately report to the SNUMC/SNUH IRB any such emergency changes for the protection of human subjects).
5. Report to the SNUMC/SNUH IRB the death, hospitalization, or serious illness of any study subject.
6. Promptly report to the SNUMC/SNUH IRB any new information that may adversely affect the safety of the subjects or the conduct of the trial.
7. Provide reports to the SNUMC/SNUH IRB concerning the progress of the research, when requested.
8. Obtain pre-approval of study advertisements from the SNUMC/SNUH IRB before use.
9. Conduct the informed consent process without coercion or undue influence, and provide the potential subject sufficient opportunity to consider whether or not to participate.

Korea FDA regulations require that the SNUMC/SNUH IRB conduct review of approved research. You will receive Continuing Review Report forms from the SNUMC/SNUH IRB. These reports must be returned even though your study may not have started.

DISTRIBUTION OF COPIES:

SPONSOR:

CRO:

OTHER:

INSTITUTION:

This is to certify that the information contained herein is true and correct as reflected in the records of the SNUMC/SNUH Institutional Review Board. **We certify that SNUMC/SNUH IRB is in full compliance with Good Clinical Practice as defined under the Korea Food and Drug Administration(KFDA) regulations and the International Conference on Harmonisation (ICH) guidelines.**

25/11//2013

King's College Hospital NHS
NHS Foundation Trust

Dr Akash Deep
Paediatric Liver
King's College Hospital
Denmark Hill
London
SE5 9RS

King's College Hospital NHS Foundation Trust
King's College Hospital
Denmark Hill
London SE5 9RS

Tel: 020 3299 9000
Fax: 020 3299 3445
Minicom: 020 3299 9009
www.kch.nhs.uk

Direct tel: 020 32991981
Direct fax: 020 3299 5515
Email: kch-tr.research@.nhs.net

Dear Dr Deep

Thank you for approaching the R&D Department with you query on analysis of routinely collected clinical data.

Analysis of routinely collected patient data does not require either REC or R&D approval so long as the analysis of identifiable patient data is undertaken by a member of the patients care team. If the analysis is to be undertaken by an individual who is not part of the patients care then the data must be anonymous.

You may apply to NRES if you would like to set up a database but this would be voluntary rather than mandatory.

Please don't hesitate to get in contact if you require any further clarification.

Regards

Will Bowen

Research Facilitator

R&D Department

THE UNIVERSITY OF IOWA

Human Subjects Office/
Institutional Review Board (IRB)
105 Hardin Library for the Health Sciences
600 Newton Road
Iowa City, Iowa 52242-1098
319-335-6564 *Fax* 319-335-7310
irb@uiowa.edu
http://research.uiowa.edu/hso

IRB ID #: 201310818

To: Patrick Brophy

From: IRB-01 DHHS Registration # IRB00000099,
Univ of Iowa, DHHS Federalwide Assurance # FWA00003007

Re: Assessment of Worldwide Acute Kidney Injury (AKI) Renal Angina and Epidemiology

Protocol Number:
Protocol Version: 1.0
Protocol Date: 10/28/13
Amendment Number/Date(s):

Approval Date: 11/05/13

Next IRB Approval Due Before: 11/05/14

Type of Application:

☒ New Project
☐ Continuing Review
☐ Modification

Type of Application Review:

☐ Full Board:
Meeting Date:
☒ Expedited

☐ Exempt

Approved for Populations:

☒ Children
☐ Prisoners
☐ Pregnant Women, Fetuses, Neonates

Source of Support:

Investigational New Drug/Biologic Name:
Investigational New Drug/Biologic Number:
Name of Sponsor who holds IND:

Investigational Device Name:
Investigational Device Number:
Sponsor who holds IDE:

This approval has been electronically signed by IRB Chair:
Catherine Woodman, MD
11/05/13 1511

IRB Approval: IRB approval indicates that this project meets the regulatory requirements for the protection of human subjects. IRB approval does not absolve the principal investigator from complying with other institutional, collegiate, or departmental policies or procedures.

Agency Notification: If this is a New Project or Continuing Review application and the project is funded by an external government or non-profit agency, the original HHS 310 form, "Protection of Human Subjects Assurance Identification/IRB Certification/Declaration of Exemption," has been forwarded to the UI Division of Sponsored Programs, 100 Gilmore Hall, for appropriate action. You will receive a signed copy from Sponsored Programs.

Recruitment/Consent: Your IRB application has been approved for recruitment of subjects not to exceed the number indicated on your application form. If you are using written informed consent, the IRB-approved and stamped Informed Consent Document(s) are attached. Please make copies from the attached "masters" for subjects to sign when agreeing to participate. The original signed Informed Consent Document should be placed in your research files. A copy of the Informed Consent Document should be given to the subject. (A copy of the *signed* Informed Consent Document should be given to the subject if your Consent contains a HIPAA authorization section.) If hospital/clinic patients are being enrolled, a copy of the IRB approved Record of Consent form should be placed in the subject's electronic medical record.

Continuing Review: Federal regulations require that the IRB re-approve research projects at intervals appropriate to the degree of risk, but no less than once per year. This process is called "continuing review." Continuing review for non-exempt research is required to occur as long as the research remains active for long-term follow-up of research subjects, even when the research is permanently closed to enrollment of new subjects and all subjects have completed all research-related interventions and to occur when the remaining research activities are limited to collection of private identifiable information. Your project "expires" at 12:01 AM on the date indicated on the preceding page ("Next IRB Approval Due on or Before"). You must obtain your next IRB approval of this project on or before that expiration date. You are responsible for submitting a Continuing Review application in sufficient time for approval before the expiration date, however the HSO will send a reminder notice approximately 60 and 30 days prior to the expiration date.

Modifications: Any change in this research project or materials must be submitted on a Modification application to the IRB for prior review and approval, except when a change is necessary to eliminate apparent immediate hazards to subjects. The investigator is required to promptly notify the IRB of any changes made without IRB approval to eliminate apparent immediate hazards to subjects using the Modification/Update Form. Modifications requiring the prior review and approval of the IRB include but are not limited to: changing the protocol or study procedures, changing investigators or funding sources, changing the Informed Consent Document, increasing the anticipated total number of subjects from what was originally approved, or adding any new materials (e.g., letters to subjects, ads, questionnaires).

Unanticipated Problems Involving Risks: You must promptly report to the IRB any serious and/or unexpected adverse experience, as defined in the UI Investigator's Guide, and any other unanticipated problems involving risks to subjects or others. The Reportable Events Form (REF) should be used for reporting to the IRB.

Audits/Record-Keeping: Your research records may be audited at any time during or after the implementation of your project. Federal and University policies require that all research records be maintained for a period of three (3) years following the close of the research project. For research that involves drugs or devices seeking FDA approval, the research records must be kept for a period of three years after the FDA has taken final action on the marketing application.

Additional Information: Complete information regarding research involving human subjects at The University of Iowa is available in the "Investigator's Guide to Human Subjects Research." Research investigators are expected to comply with these policies and procedures, and to be familiar with the University's Federalwide Assurance, the Belmont Report, 45CFR46, and other applicable regulations prior to conducting the research. These documents and IRB application and related forms are available on the Human Subjects Office website or are available by calling 335-6564.

FOR IRB USE ONLY
APPROVED BY: IRB-01
IRB ID #: 201310818
APPROVAL DATE: 11/05/13
EXPIRATION DATE: 11/05/14

INFORMED CONSENT DOCUMENT

Project Title: AWARE – Assessment of Worldwide Acute Kidney Injury (AKI), Renal Angina, and Epidemiology

Principal Investigator: Patrick Brophy, MD

Research Team Contact: Doug Russo
319-467-5109

- If you are the parent/guardian of a child under 18 years old who is being invited to be in this study, the word "you" in this document refers to your child. You will be asked to read and sign this document to give permission for your child to participate.
- If you are a teenager reading this document because you are being invited to be in this study, the word "you" in this document refers to you. You will be asked to read and sign this document to indicate your willingness to participate.

This consent form describes the research study to help you decide if you want to participate. This form provides important information about what you will be asked to do during the study, about the risks and benefits of the study, and about your rights as a research subject.

- If you have any questions about or do not understand something in this form, you should ask the research team for more information.
- You should discuss your participation with anyone you choose such as family or friends.
- Do not agree to participate in this study unless the research team has answered your questions and you decide that you want to be part of this study.

WHAT IS THE PURPOSE OF THIS STUDY?

This is a research study. We are inviting you to participate in this research study because you are between the ages of 90 days and 25 years of age and are currently admitted to a pediatric intensive care unit.

The purpose of this research study is to learn about the causes and outcomes of acute kidney injury (AKI) in children that have been admitted to pediatric intensive care units (PICU) and pediatric cardiac intensive care units (CICU).

HOW MANY PEOPLE WILL PARTICIPATE?

Approximately 185 people will take part in this study conducted by investigators at the University of Iowa. There are 30 participating sites and therefore we are expecting a total of 5,550 children from all sites.

HOW LONG WILL I BE IN THIS STUDY?

If you agree to take part in this study, your involvement will last as long as you are in the PICU or CICU up to 28 days depending on the length of your stay. If you are in the PICU or CICU for more than 2 days and are transferred to the general hospital, we will follow you for up to 28 days. If you are discharged from the PICU or CICU in less than 2 days, you will be withdrawn from the study and we will not use your data.

WHAT WILL HAPPEN DURING THIS STUDY?

The consent for will be reviewed with you by a member of the research team. You will have the opportunity to ask any questions you may have about the study. If you are willing to participate, you will be asked to sign this consent form. You will be given a copy of this signed consent form to keep for your records. The research staff will review your medical history and demographic information.
For this study, we will need to collect your protected health information (PHI) from your medical records and research records. The types of information that will be used and shared from these records include:

- Laboratory test results, diagnosis, and medications
- Reports and notes from clinical and research observations
- Imaging (like CT scans, MRI scans, x-rays, etc.) studies and reports
- If applicable, information concerning HIV testing or the treatment of AIDS and AIDS-related conditions, drug or alcohol abuse, drug-related conditions, alcoholism, and/or psychiatric/psychological conditions (but not psychotherapy notes)

Data Collection: Your clinical data will be collected from admission to the PICU or CICU through the first seven days and up to 28 days from your general hospital admission. If you are discharged from the PICU at less than 48 hours, you will be withdrawn from the study and any patient data that was previously collected will eliminated and thus, not analyzed.
Urine Collection (optional): Only available if you are admitted to the PICU or CICU who have urinary drainage system in place. Please mark either Yes or No to the collection of urine samples for yourself and sign below.

☐ I agree to the collection of urine samples
☐ I do not agree to the collection of urine samples

Subject Signature: ______________________________

FOR IRB USE ONLY<br>APPROVED BY: IRB-01<br>IRB ID #: 201310818<br>APPROVAL DATE: 11/05/13<br>EXPIRATION DATE: 11/05/14

If subject is less than 18 years old:

☐ I agree to the collection of my child's urine samples
☐ I do not agree to the collection of my child's urine samples

Parent Signature: ______________________________

Urine/Data Storage for Future Use
As part of this study, we are obtaining data and urine samples from you. We would like to study your urine sample and data in the future, after this study is over.

The tests we and others might want to use to study your data and urine samples may not even exist as this time. Therefore, we are asking for your permission to store your data and urine samples so that we can study them in the future. These future studies may provide additional information that will be helpful in understanding the causes and outcomes of acute kidney injury (AKI) but it is unlikely that what we learn from these studies will have a direct benefit to you. It is possible that your data and urine samples might be used to develop products or tests that could be patented and licensed. There are no plans to provide financial compensation to you should this occur.

Your urine samples will be stored up to five years *with* an ID code found on the samples. Although an ID code could seemingly be used to identify samples to individuals, the identifier information will be kept separate and only the research group will have access to the identifier information.
Therefore, it would not be possible for the ID code found on samples to be traced back to the individual.

My urine samples may be stored/shared for future use.

_____ Yes   _____ No

WHAT ARE THE RISKS OF THIS STUDY?
You may experience one or more of the risks indicated below from being in this study. In addition to these, there may be other unknown risks, or risks that we did not anticipate, associated with being in this study.
There are no physical risks associated with this study. There is a potential risk of loss of confidentiality. Measures in place to protect your confidentiality are indicated in the "What About Confidentiality" section later in this document.

WHAT ARE THE BENEFITS OF THIS STUDY?

You will not benefit from being in this study.

However, we hope that, in the future, other people might benefit from this study because we may learn more about the patterns, causes, and effects of acute kidney injury. This may help other children with acute kidney injury later on.

WILL IT COST ME ANYTHING TO BE IN THIS STUDY?

You will not have any costs for being in this research study.

WILL I BE PAID FOR PARTICIPATING?

You will not be paid for being in this research study.

WHO IS FUNDING THIS STUDY?

The University and the research team are receiving no payments from other agencies, organizations, or companies to conduct this research study.

WHAT ABOUT CONFIDENTIALITY?

We will keep your participation in this research study confidential to the extent permitted by law. However, it is possible that other people such as those indicated below may become aware of your participation in this study and may inspect and copy records pertaining to this research. Some of these records could contain information that personally identifies you.

- federal government regulatory agencies,
- auditing departments of the University of Iowa, and
- the University of Iowa Institutional Review Board (a committee that reviews and approves research studies)

To help protect your confidentiality, electronic data collected for study purposes will be stored separate from all personal health information in a locked office on a password protected network drive. A key with the patient's name medical record number, date of birth, and study ID will be kept separately from the study data. Paper/hard copies will be kept in locked file cabinets located in the primary researcher's office. Only the research team will have access both the paper/hard copy and electronic records. Until the urine samples are shipped to The Center for Acute Care Nephrology Biomarker Core Laboratory in the Division of Nephrology and Hypertension at Cincinnati Children's Hospital Medical Center, the samples will be stored in the laboratory of Dr. Patrick Brophy. Only the research team will have access to these biological samples. If we write a report or article about this study or share the study data set with others, we will do so in such a way that you cannot be directly identified.

The University of Iowa Hospitals and Clinics generally requires that we document in your medical record chart that you are participating in this study. The information included in the chart will provide contact information for the research team as well as information about the risks associated with this study. We will keep this Informed Consent Document in our research files; it will not be placed in your medical record chart.

WILL MY HEALTH INFORMATION BE USED DURING THIS STUDY?

The Federal Health Insurance Portability and Accountability Act (HIPAA) requires your health care provider to obtain your permission for the research team to access or create "protected health information" about you for purposes of this research study. Protected health information is information that personally identifies you and relates to your past, present, or future physical or mental health condition or care. We will access or create health information about you, as described in this document, for purposes of this research study. Once your health care provider has disclosed your protected health information to us, it may no longer be protected by the Federal HIPAA privacy regulations, but we will continue to protect your confidentiality as described under "Confidentiality."

We may share your health information related to this study with other parties including federal government regulatory agencies, the University of Iowa Institutional Review Boards and support staff.

You cannot participate in this study unless you permit us to use your protected health information. If you choose *not* to allow us to use your protected health information, we will discuss any non-research alternatives available to you. Your decision will not affect your right to medical care that is not research-related. Your signature on this Consent Document authorizes your health care provider to give us permission to use or create health information about you.

Although you may not be allowed to see study information until after this study is over, you may be given access to your health care records by contacting your health care provider. Your permission for us to access or create protected health information about you for purposes of this study has no expiration date. You may withdraw your permission for us to use your health information for this research study by sending a written notice to **Dr. Patrick Brophy, 1269A CBRB 200 Hawkins Drive Iowa City, IA, 52242.** However, we may still use your health information that was collected before withdrawing your permission. Also, if we have sent your health information to a third party, such as the study sponsor, or we have removed your identifying information, it may not be possible to prevent its future use. You will receive a copy of this signed document.

IS BEING IN THIS STUDY VOLUNTARY?

Taking part in this research study is completely voluntary. You may choose not to take part at all. If you decide to be in this study, you may stop participating at any time. If you decide not to be in this study, or if you stop participating at any time, you won't be penalized or lose any benefits for which you otherwise qualify.

What if I Decide to Drop Out of the Study?

You may choose to withdraw your permission at any time. A withdrawal of your permission to use and share your Protected Health Information (PHI) would also include a withdrawal from participation in the research study. If you wish to withdraw your permission to use and share your PHI you need to notify the study investigator. Your request will be effective immediately and no further PHI about you will be collected for research analysis. PHI already collected can continue to be used and shared.

Will I Receive New Information About the Study while Participating?
If we obtain any new information during this study that might affect your willingness to continue participating in the study, we'll promptly provide you with that information.

WHAT IF I HAVE QUESTIONS?

We encourage you to ask questions. If you have any questions about the research study itself, please contact: **Doug Russo at 319-467-5109** If you experience a research-related injury, please contact: **Dr. Patrick Brophy at 319-356-1334**.
If you have questions, concerns, or complaints about your rights as a research subject or about research related injury, please contact the Human Subjects Office, 105 Hardin Library for the Health Sciences, 600 Newton Rd, The University of Iowa, Iowa City, IA 52242-1098, (319) 335-6564, or e-mail irb@uiowa.edu. General information about being a research subject can be found by clicking "Info for Public" on the Human Subjects Office web site, http://hso.research.uiowa.edu/. To offer input about your experiences as a research subject or to speak to someone other than the research staff, call the Human Subjects Office at the number above.

This Informed Consent Document is not a contract. It is a written explanation of what will happen during the study if you decide to participate. You are not waiving any legal rights by signing this Informed Consent Document. Your signature indicates that this research study has been explained to you, that your questions have been answered, and that you agree to take part in this study. You will receive a copy of this form.

Subject's Name (printed): ____

Do not sign this form if today's date is on or after EXPIRATION DATE: 11/05/14.

______________________________ ______________________

(Signature of Subject) (Date)

FOR IRB USE ONLY
APPROVED BY: IRB-01
IRB ID #: 201310818
APPROVAL DATE: 11/05/13
EXPIRATION DATE: 11/05/14

Parent/Guardian's Name and Relationship to Subject:

(Name - printed)

(Relationship to Subject - printed)

Do not sign this form if today's date is on or after EXPIRATION DATE: 11/05/14.

(Signature of Parent/Guardian)

(Date)

Statement of Person Who Obtained Consent

I have discussed the above points with the subject or, where appropriate, with the subject's legally authorized representative. It is my opinion that the subject understands the risks, benefits, and procedures involved with participation in this research study.

(Signature of Person who Obtained Consent)

(Date)

Yale University

Human Investigation Committee
55 College Street
New Haven CT, 06510

Telephone: 203-785-4688
Fax: 203-785-2847
http://info.med.yale.edu/hic

**To:**	Edward Faustino, M.D.
**From:**	The Human Investigation Committee
**Date:**	11/22/2013
**HIC Protocol #:**	1311013054
**Study Title:**	Assessment of worldwide acute kidney injury, renal angina and epidemiology (The AWARE Study)
**Committee Action**	**Expedited Approval**
**HIC Action Date:**	11/22/2013
**Approval Date:**	11/22/2013
**Expiration Date:**	11/21/2014
**Submission Type:**	**Initial Application**

Your request regarding the above-referenced protocol has been APPROVED following an expedited review by the Human Investigation Committee. This review meets approval criteria set forth in 45 CFR 46.111. The approval period for this protocol is considered to be the Approval Date through the Expiration Date.

Review Comments:

- The HIC found this study to meet the requirements of 45 CFR § 46.404 in that it presents no more than minimal risk to the minor subjects.

- The Committee finds that informed consent can be waived for this study per federal regulation 45 CFR 46.116(d). This part of the regulations states that 1) this research involves no more than minimal risk to the subjects, 2) the waiver or alteration will not adversely affect the rights and welfare of the subjects, 3) the research could not practicably be carried out without the waiver and 4) whenever appropriate the subjects will be provided with additional pertinent information after participation.

- Consistent with the provisions for waiver of informed consent contained in 45 CFR § 46.116, the HIC approves the waiver of parental permission as per 45 CFR § 46.408 in that the research protocol is designed for conditions or for a subject population for which parental or guardian permission is not a reasonable requirement to protect the subjects and an appropriate mechanism for protecting the children who will participate as subjects in the research is substituted, and provided further that the waiver is not inconsistent with federal, state or local law.
  The HIC has determined that assent from the children may be waived in accordance with § 46.116 of subpart A.

- Approved and uploaded into COEUS is the HIC protocol application.

- This review reflects sponsor protocol version 1.0, dated 28Oct2013.

- A HIPAA waiver has been approved via expedited review for access to and use o fmedical record information without obtaining written approval ("authorization") from the subject for the use of the data. This waiver does not authorize subject contact.

The IRB finds that the use or disclosure of protected health information involves no more than a minimal risk to the privacy of individuals, based on (1) an adequate plan to protect the identifiers from improper use and disclosure; (2) an adequate plan to destroy the identifiers at the earliest opportunity consistent with conduct of the research, unless there is a health or research justification for retaining the identifiers or such retention is otherwise required by law; and (3) adequate written assurances that the protected health information will not be reused or disclosed to any other person or entity, except as required by law, for authorized oversight of the research study, or for other research for which the use or disclosure of protected health information would be permitted by this subpart;

The IRB also finds that the research could not practicably be conducted without the waiver or alteration; and the research could not practicably be conducted without access to and use or the protected health information.

HIPAA regulations require that accounting logs be maintained when researchers access patient records under a waiver of authorization including those approved for recruitment purposes. You are thereby reminded of your obligation to create the log. A spreadsheet is available on the HRPP web site to assist in the collection of accounting log information. These logs must be forwarded to the HIPAA Privacy Office – hipaa@yale.edu. For further information on the accounting of disclosures, please see http://www.yale.edu/ppdev/policy/5032/5032.pdf

It is the investigator's responsibility to obtain reapproval of ongoing research prior to the Expiration Date. Please submit the request for reapproval at least two months prior to the expiration date to allow for reapproval processing and review.

Adverse Reactions: Serious, unanticipated, and related adverse events, and unanticipated problems involving risk to subjects or others must be reported within 48 hours to the HIC, using Form 6A.

Amendments: If you wish to change any aspect of this study, such as the study procedures or processes, the informed consent document(s), recruitment activities, or wish to add or remove investigators or key study personnel, you must communicate your requested changes to the HIC using the appropriate form located at http://www.yale.edu/hrpp. Any changes must be approved by the HIC prior to implementation.

Request to Close: When the study procedures and the data analysis are fully complete, the Form #5C must be completed and sent to the HIC requesting that the study be closed. Investigators should attach a copy of the study findings. Abstracts or publications satisfy this findings requirement.

Please keep this memo with your copy of the approved protocol.

Vanderbilt University
Institutional Review Board
504 Oxford House Nashville, Tennessee 37232-4315
(615) 322-2918 Fax: (615) 343-2648
www.mc.vanderbilt.edu/irb

December 27, 2013

Geoffrey Fleming, M.D.
Pediatrics
5121 DOT 9075

RE: IRB# 131921 "Assessment of Worldwide AKI, Renal Angina and Epidemiology"

Dear Geoffrey Fleming, M.D.:

A sub-committee of the Institutional Review Board reviewed the research application identified above. The sub-committee determined the study poses minimal risk to participants, and the application is approved under 45 CFR 46.110 (F)(5). Approval is extended for the Protocol version 1.0 dated 10/28/2013. The informed consent process is waived in accordance with 45 CFR 46.116 (d).

As the Principal Investigator, you are responsible for the accurate documentation, investigation and follow-up of all possible study-related adverse events and unanticipated problems involving risks to participants or others. The IRB Adverse Event reporting policy III.G is located on the IRB website at http://www.mc.vanderbilt.edu/irb/.

If this trial requires registration as a clinical trial, accrual cannot begin until this study has been registered at clinicaltrials.gov and a National Clinical Trial Number (NCT) provided. Please provide the NCT# to the IRB as soon as it is obtained. If an approval is required from an additional source other than the Vanderbilt IRB, this must be obtained prior to study initiation. These approvals may include, but are not limited to CRC, SRC, IND, IDE.

Please note that approval is for a 12-month period. Any changes to the research study must be presented to the IRB for approval prior to implementation.

DATE OF IRB APPROVAL: 12/27/2013 **DATE OF IRB EXPIRATION: 12/26/2014**

Sincerely,

Saralyn R. Williams

Saralyn R. Williams, M.D., Vice-Chair
Institutional Review Board
Health Sciences Committee #3

SRW/ag
Electronic Signature: Saralyn R Williams/VUMC/Vanderbilt : (84AAD8CEB7F3CC8D2F62D1AB9EBB2394)
Signed On: 12/27/2013 03:45:44 PM CST

V I R G I N I A C O M M O N W E A L T H U N I V E R S I T Y

VCU

Make it real.

Office of Research
Office of Research Subjects Protection
BioTechnology Research Park
800 East Leigh Street, Suite 3000
P.O. Box 980568
Richmond, Virginia 23298-0568

(804) 828-0868
Fax: (804) 827-1448

TO: Duane Williams
Timothy Bunchman
Sabrina Minter
Duane Williams
Melissa Yopp

CC:

FROM: VCU IRB Panel C

RE: Duane Williams ; IRB HM20000679 Assessment of Worldwide AKI, Renal Angina and Epidemiology (AWARE)

On April 7, 2014, the referenced research study was ***approved*** by expedited review according to 45 CFR 46.110 categories 3 and 5 by VCU IRB Panel C.

This study involves children, and is approved under children's category 45 CFR 46.404.

45 CFR 46.404	Research involving no greater than minimal risk to children, with adequate provisions for soliciting the assent of the children and permission of their parents or guardians, as set forth in Sec. 46.408

- Note: For children involved in this study, the IRB finds that **the permission and signature of one parent is required**.

The information found in the electronic version of this study's smart form and uploaded documents now represents the currently approved study, documents, informed consent process, and HIPAA pathway (if applicable). You may access this information by clicking the Study Number above.

This approval expires on March 31, 2015. Federal Regulations/VCU Policy and Procedures require continuing review prior to continuation of approval past that date. Continuing Review notices will be sent to you prior to the scheduled review.

If you have any questions, please contact the Office of Research Subjects Protection (ORSP) or the IRB reviewer(s) assigned to this study. The reviewer(s) assigned to your study will be listed in the History tab and on the study workspace. Click on their name to see their contact information.

Attachment – Conditions of Approval

Conditions of Approval:

In order to comply with federal regulations, industry standards, and the terms of this approval, the investigator must (as applicable):

1. Conduct the research as described in and required by the Protocol.
2. Obtain informed consent from all subjects without coercion or undue influence, and provide the potential subject sufficient opportunity to consider whether or not to participate (unless Waiver of Consent is specifically approved or research is exempt).
3. Document informed consent using only the most recently dated consent form bearing the VCU IRB "APPROVED" stamp (unless Waiver of Consent is specifically approved).
4. Provide non-English speaking patients with a translation of the approved Consent Form in the research participant's first language. The Panel must approve the translated version.
5. Obtain prior approval from VCU IRB before implementing any changes whatsoever in the approved protocol or consent form, unless such changes are necessary to protect the safety of human research participants (e.g., permanent/temporary change of PI, addition of performance/collaborative sites, request to include newly incarcerated participants or participants that are wards of the state, addition/deletion of participant groups, etc.). Any departure from these approved documents must be reported to the VCU IRB immediately as an Unanticipated Problem (see #7).
6. Monitor all problems (anticipated and unanticipated) associated with risk to research participants or others.
7. Report Unanticipated Problems (UPs), including protocol deviations, following the VCU IRB requirements and timelines detailed in VCU IRB WPP VIII-7:
8. Obtain prior approval from the VCU IRB before use of any advertisement or other material for recruitment of research participants.
9. Promptly report and/or respond to all inquiries by the VCU IRB concerning the conduct of the approved research when so requested.
10. All protocols that administer acute medical treatment to human research participants must have an emergency preparedness plan. Please refer to VCU guidance on http:/www.research.vcu.edu/irb/guidance.htm.
11. The VCU IRBs operate under the regulatory authorities as described within:
    a. U.S. Department of Health and Human Services Title 45 CFR 46, Subparts A, B, C, and D (for all research, regardless of source of funding) and related guidance documents.
    b. U.S. Food and Drug Administration Chapter I of Title 21 CFR 50 and 56 (for FDA regulated research only) and related guidance documents.
    c. Commonwealth of Virginia Code of Virginia 32.1 Chapter 5.1 Human Research (for all research).

Yale University

Human Investigation Committee
55 College Street
New Haven CT, 06510

Telephone: 203-785-4688
Fax: 203-785-2847
http://info.med.yale.edu/hic

**To:**	Edward Faustino, M.D.
**From:**	The Human Investigation Committee
**Date:**	11/22/2013
**HIC Protocol #:**	1311013054
**Study Title:**	Assessment of worldwide acute kidney injury, renal angina and epidemiology (The AWARE Study)
**Committee Action**	**Expedited Approval**
**HIC Action Date:**	11/22/2013
**Approval Date:**	11/22/2013
**Expiration Date:**	11/21/2014
**Submission Type:**	**Initial Application**

Your request regarding the above-referenced protocol has been APPROVED following an expedited review by the Human Investigation Committee. This review meets approval criteria set forth in 45 CFR 46.111. The approval period for this protocol is considered to be the Approval Date through the Expiration Date.

Review Comments:

- The HIC found this study to meet the requirements of 45 CFR § 46.404 in that it presents no more than minimal risk to the minor subjects.

- The Committee finds that informed consent can be waived for this study per federal regulation 45 CFR 46.116(d). This part of the regulations states that 1) this research involves no more than minimal risk to the subjects, 2) the waiver or alteration will not adversely affect the rights and welfare of the subjects, 3) the research could not practicably be carried out without the waiver and 4) whenever appropriate the subjects will be provided with additional pertinent information after participation.

- Consistent with the provisions for waiver of informed consent contained in 45 CFR § 46.116, the HIC approves the waiver of parental permission as per 45 CFR § 46.408 in that the research protocol is designed for conditions or for a subject population for which parental or guardian permission is not a reasonable requirement to protect the subjects and an appropriate mechanism for protecting the children who will participate as subjects in the research is substituted, and provided further that the waiver is not inconsistent with federal, state or local law.
  The HIC has determined that assent from the children may be waived in accordance with § 46.116 of subpart A.

- Approved and uploaded into COEUS is the HIC protocol application.

- This review reflects sponsor protocol version 1.0, dated 28Oct2013.

- A HIPAA waiver has been approved via expedited review for access to and use o fmedical record information without obtaining written approval ("authorization") from the subject for the use of the data. This waiver does not authorize subject contact.

The IRB finds that the use or disclosure of protected health information involves no more than a minimal risk to the privacy of individuals, based on (1) an adequate plan to protect the identifiers from improper use and disclosure; (2) an adequate plan to destroy the identifiers at the earliest opportunity consistent with conduct of the research, unless there is a health or research justification for retaining the identifiers or such retention is otherwise required by law; and (3) adequate written assurances that the protected health information will not be reused or disclosed to any other person or entity, except as required by law, for authorized oversight of the research study, or for other research for which the use or disclosure of protected health information would be permitted by this subpart;

The IRB also finds that the research could not practicably be conducted without the waiver or alteration; and the research could not practicably be conducted without access to and use or the protected health information.

HIPAA regulations require that accounting logs be maintained when researchers access patient records under a waiver of authorization including those approved for recruitment purposes. You are thereby reminded of your obligation to create the log. A spreadsheet is available on the HRPP web site to assist in the collection of accounting log information. These logs must be forwarded to the HIPAA Privacy Office – hipaa@yale.edu. For further information on the accounting of disclosures, please see http://www.yale.edu/ppdev/policy/5032/5032.pdf

It is the investigator's responsibility to obtain reapproval of ongoing research prior to the Expiration Date. Please submit the request for reapproval at least two months prior to the expiration date to allow for reapproval processing and review.

Adverse Reactions: Serious, unanticipated, and related adverse events, and unanticipated problems involving risk to subjects or others must be reported within 48 hours to the HIC, using Form 6A.

Amendments: If you wish to change any aspect of this study, such as the study procedures or processes, the informed consent document(s), recruitment activities, or wish to add or remove investigators or key study personnel, you must communicate your requested changes to the HIC using the appropriate form located at http://www.yale.edu/hrpp. Any changes must be approved by the HIC prior to implementation.

Request to Close: When the study procedures and the data analysis are fully complete, the Form #5C must be completed and sent to the HIC requesting that the study be closed. Investigators should attach a copy of the study findings. Abstracts or publications satisfy this findings requirement.

Please keep this memo with your copy of the approved protocol.
